# Distribution and correlation between phylogeny and functional traits of cowpea (*Vigna unguiculata* L. Walp.)-nodulating microsymbionts from Ghana and South Africa

**DOI:** 10.1038/s41598-018-36324-0

**Published:** 2018-12-20

**Authors:** Mustapha Mohammed, Sanjay K. Jaiswal, Felix D. Dakora

**Affiliations:** 1 Department of Crop Sciences, Tshwane University of Technology, Private Bag X680, Pretoria, 0001 South Africa; 2 Chemistry Department, Tshwane University of Technology, Private Bag X680, Pretoria, 0001 South Africa

## Abstract

Cowpea (*Vigna unguiculata* L. Walp.) is indigenous to Africa, and highly valued for its N_2_-fixing trait and the nutritional attributes of its grain and leaves. The species’ ability to establish effective symbiosis with diverse rhizobial populations gives it survival and growth advantage in N-limited environments. To explore the functional diversity and phylogenetic positions of rhizobia nodulating cowpea in Africa, nodules were collected from various cowpea varieties grown in soils from the Guinea savanna and Sudano-sahelian agroecologies of Northern Ghana, and from the lowveld and middleveld areas of Mpumalanga Province in South Africa. Box-PCR profiling and multilocus sequence analysis revealed the presence of diverse microsymbionts responsible for cowpea nodulation across the study sites. BOX-PCR amplifications yielded variable band sizes, ranging from 618 bp to 5354 bp, which placed the isolates in six major clusters (Cluster A–F). Phylogenetic analysis based on 16S rRNA, *atpD*, *glnII*, *gyrB*, *rpoB*, *nifH* and *nodC* genes revealed the presence of diverse *Bradyrhizobium* sp. closely related to *Bradyrhizobium daqingense, Bradyrhizobium subterraneum, Bradyrhizobium yuanmingense, Bradyrhizobium embrapense, Bradyrhizobium pachyrhizi, Bradyrhizobium elkanii* and novel *Bradyrhizobium* species in the soils studied, a finding that could be attributed to the unique edapho-climatic conditions of the contrasting environments. The test isolates exhibited distinct symbiotic efficiencies, and also induced variable (p ≤ 0.001) photosynthetic rates, leaf transpiration, total chlorophyll and shoot biomass accumulation on cowpea (their homologous host). Canonical correspondence analysis showed that the distribution of these microsymbionts was influenced by the concentrations of macro- and micronutrients in soils. The pairwise genetic distances derived from phylogenies and nodule functioning showed significant (p < 0.05) correlation, which suggests that local environmental factors played a major role in the cowpea-*Bradyrhizobium* symbiosis.

## Introduction

Legumes constitute an important component of sustainable agriculture and ecosystem functioning as a result of their contribution to soil health from symbiosis with nodule microsymbionts^1,2^. In Africa, grain legumes such as cowpea (*Vigna unguiculata* L. Walp.) are highly valued for their N_2_-fixing ability and the nutritional attributes of their grains and leaves, both of which are important for sustainable farming systems and household food security^3^. The grain of cowpea can contain up to 25% protein which is rich in lysine, a limiting essential S-containing amino acid in cereals^4,5^. Most cowpea genotypes tend to accumulate greater levels of micro- and macro-nutrients in their leaves when compared to the grain, a trait that is useful for combating micro-nutrient deficiency among rural households, where cowpea leaves are first harvested as food before the grain^3^.

Cowpea is indigenous to Africa, with both wild and cultivated forms found across the continent. Although the exact centre of origin or diversification is still uncertain^6^, a west African centre of cowpea domestication is supported by the oldest archaeological evidence in the Kintampo rock shelter in central Ghana^7,8^. A greater proportion (96.4%) of the world’s cowpea is produced in Africa, with Nigeria and Niger together accounting for 72% of the global production in 2016, while the Americas, Asia and Europe accounted for 1.2%, 2% and 0.4%, respectively, of the world’s production^9^. Despite the importance of cowpea to the livelihoods of rural communities, its average grain yield on farmers’ fields is low (typically < 1 t.ha^−1^) due to a range of factors which include soil nutrient deficiencies, sub-optimal symbiosis, and insect pests diseases. However, yields of up to 2.5 t.ha^−1^ are attainable depending on the genotype, location and cultivation practices used^10–12^.

Like most grain legumes, cowpea can establish effective symbiosis with diverse rhizobial populations belonging to the genera *Bradyrhizobium* and *Rhizobium*^10,13^, which gives this legume an ability to grow in diverse environments where other legumes may fail to survive. The symbiotic relationship between legumes and rhizobia commences via an intricate exchange of molecular signals between the two partners, and sustained by mutual exchange of photosynthate and nitrogenous solutes between the legume and the bacterial symbionts^14^. As a result, increased nitrogen fixation in root nodules generally drives plant growth through increased photosynthetic rates due to the greater C sink strength of nodulated roots^15,16^. The cowpea-rhizobia symbiosis can account for up to 96% of the plant’s N requirement, as well as contribute to the N needs of subsequent cereal crops in Sub-Saharan Africa, where the soils are nutrient-poor^17,18^.

There is new evidence that the distribution and biodiversity of legume microsymbionts are shaped by geographical location and the soil’s physico-chemical properties^10,19^. Despite the agronomic, ecological and environmental significance of the legume-rhizobia symbiosis, the process is influenced by several factors which include the nature of the legume host and the rhizobial symbiont^16^. The identification of novel root-nodule bacteria and their evaluation for symbiotic effectiveness therefore forms the basis for their use in inoculant formulation aimed at tapping the agronomic and ecological benefits of the legume-rhizobia symbiosis^20^.

Although earlier studies have identified *Bradyrhizobium* as the microsymbiont nodulating cowpea in Botswana, Ghana, South Africa^21^, Senegal^10^, Mozambique^13^, Ethiopia^22^, Greece^23^, Spain^24^ and Brazil^25,26^, much remains to be known about the diversity and phylogenetic position of cowpea microsymbionts in Africa, the centre of its diversification. Characterization of rhizobia native to different geographic locations in Africa is likely to also improve our understanding of their contribution to overall ecosystem function, and help to unravel the factors shaping microsymbiont diversity and distribution in soils.

Genomic fingerprinting techniques such as rep-PCR (BOX-PCR) have been used to assess genetic diversity among populations of rhizobia, including those that share close relatedness^13^. Although sequence analysis of the 16S rRNA gene is also useful in establishing the phylogenies of legume microsymbionts^27,28^, the multilocus sequence analysis is often used in combination with the 16S rRNA due to the lower resolving power of this gene^19,23^. Careful selection and sequencing of 3 to 5 housekeeping or protein-coding genes, in addition to the 16S rRNA gene, yielded reliable phylograms in earlier studies^23,27^. However, the diversity of microsymbionts based on either trait, or phylogenetic information, was unclear at the level of community analysis^29^. Therefore, use of the two approaches has been suggested for microsymbiont differentiation^30^. The aim of this study was to assess the phylogeny, functional diversity and distribution of cowpea microsymbionts originating from different locations within the Guinea Savanna and the Sudano-sahelian Savanna of Ghana, and from Nelspruit and Klipplaatdrift in the lowveld and middleveld areas of Mpumalanga Province in South Africa.

## Results

### Nodulation and colony characteristics

A total of 98 bacterial isolates were obtained from the root nodules collected from cowpea. Of these, only 54 isolates (53%) could form root nodules on cowpea (the homologous host) under glasshouse conditions. Except for TUTVUSA35, TUTVUGH5, TUTVUGH4 and TUTVUGH1, the colonies of all isolates were visible after 5 d of incubation on YMA plates. Colonies of isolate TUTVUSA49 obtained from Kliplaadrift were visible on YMA plates after 12 days of incubation. Colony sizes of all the isolates varied from 1 to 4 mm in diameter (Table 1).

**Table 1:** Geographical origin (country and location), host genotype and morphological description (size, shape, colour and appearance/opacity) of cowpea isolates used in this study.

Isolates	Country	location	Cluster (≥70%)	Major Cluster	Genotype	Growth	Size (mm)	shape	colour	Appearance
TUTVUGH9	Ghana	Damongo	1	A	IT90K-277-2	5	2	round	watery	Translucent
TUTVUGH20	Ghana	Garu	2		Apagbaala	5	3	Round	Cream	Opaque
TUTVUSA44	South Africa	Klipplaatdrift	3		Nhyira	9	1	Round	Watery	Translucent
TUTVUGH25	Ghana	Garu	4		Songotra	6	1	Round	Cream	Opaque
TUTVUGH16	Ghana	Damongo	5	B	Omandaw	5	2	Round	Watery	Translucent
TUTVUGH17	Ghana	Damongo	6		Padi-tuya	5	3	Oval	Watery	Translucent
TUTVUGH7	Ghana	Damongo	7		Padi-tuya	5	3	Round	Cream	Opaque
TUTVUGH11	Ghana	Damongo	7		Apagbaala	5	2	Round	Watery	Translucent
TUTVUGH15	Ghana	Damongo	7		IT90K-277-2	5	4	Oval	Watery	Transparent
TUTVUGH6	Ghana	Damongo	8		Songotra	5	3	Round	Watery	Translucent
TUTVUGH8	Ghana	Damongo	9		IT90K-277-2	5	3	Round	Watery	Translucent
TUTVUGH12	Ghana	Damongo	9		Songotra	5	3	Oval	Watery	Translucent
TUTVUGH13	Ghana	Damongo	9		IT90K-277-2	5	1	Round	Watery	Translucent
TUTVUGH18	Ghana	Garu	10		Apagbaala	5	2	Oval	Watery	Translucent
TUTVUSA51	South Africa	Klipplaatdrift	11		Nhyira	7	<1	Oval	Cream	Opaque
TUTVUSA53	South Africa	Klipplaatdrift	12		Bawutawuta	7	1	Round	Cream	Opaque
TUTVUSA54	South Africa	Klipplaatdrift	13		Apagbaala	6	2	Round	Cream	Opaque
TUTVUSA55	South Africa	Klipplaatdrift	14		Bawutawuta	7	<1	Round	Cream	Opaque
TUTVUGH10	Ghana	Damongo	15		Omandaw	6	3	Oval	Watery	Transparent
TUTVUGH14	Ghana	Damongo	15		Songotra	5	3	Oval	Watery	Transparent
TUTVUGH24	Ghana	Garu	16		Songotra	5	2	Round	Watery	Translucent
TUTVUGH21	Ghana	Garu	17		IT90K-277-2	5	2	Round	Watery	Translucent
TUTVUGH22	Ghana	Garu	17		Omandaw	5	3	Oval	Watery	Translucent
TUTVUSA50	South Africa	Klipplaatdrift	18		Padi-tuya	9	2	Oval	Cream	Opaque
TUTVUSA56	South Africa	Klipplaatdrift	19		Bawutawuta	9	<1	Round	Cream	Opaque
TUTVUSA34	South Africa	Nelspruit	20		Songotra	5	2	Round	Watery	Translucent
TUTVUSA36	South Africa	Nelspruit	20		IT90K-277-2	5	3	Oval	Watery	Transparent
TUTVUSA33	South Africa	Nelspruit	21		Songotra	5	2	Irregular	Watery	Translucent
TUTVUSA35	South Africa	Nelspruit	22		IT90K-277-2	3	2	Round	Watery	Translucent
TUTVUSA47	South Africa	Klipplaatdrift	23		Omandaw	5	1	Round	Cream	Opaque
TUTVUSA48	South Africa	Klipplaatdrift	24		Omandaw	7	<1	Round	Cream	Opaque
TUTVUGH5	Ghana	Nyankpala	25		Padi-tuya	4	3	Round	Watery	Translucent
TUTVUGH19	Ghana	Garu	26		Padi-tuya	5	2	Round	Watery	Transparent
TUTVUGH23	Ghana	Garu	27		Padi-tuya	5	2	Round	Watery	Translucent
TUTVUSA37	South Africa	Klipplaatdrift	28	C	Omandaw	7	1	Round	Cream	Opaque
TUTVUSA43	South Africa	Klipplaatdrift	29		Omandaw	7	<1	Round	Cream	Opaque
TUTVUSA46	South Africa	Klipplaatdrift	30		Padi-tuya	7	1	Round	Cream	Opaque
TUTVUSA40	South Africa	Klipplaatdrift	31		Nhyira	9	1	Round	Cream	Opaque
TUTVUSA42	South Africa	Klipplaatdrift	31		Bawutawuta	9	<1	Round	Cream	Opaque
TUTVUGH26	Ghana	Garu	32		IT90K-277-2	6	1	Round	Watery	Translucent
TUTVUSA49	South Africa	Klipplaatdrift	33		Bawutawuta	12	2	Round	Cream	Opaque
TUTVUSA38	South Africa	Klipplaatdrift	34		Nhyira	9	<1	Round	Cream	Opaque
TUTVUSA41	South Africa	Klipplaatdrift	35		Omandaw	9	2	Round	Watery	Translucent
TUTVUSA45	South Africa	Klipplaatdrift	36		Nhyira	9	1	Round	Cream	Opaque
TUTVUGH27	Ghana	Garu	37	D	Omandaw	6	2	Round	Cream	Opaque
TUTVUSA28	South Africa	Nelspruit	38		IT90K-277-2	6	2	Round	Watery	Translucent
TUTVUGH2	Ghana	Nyankpala	39	E	Songotra	5	3	Oval	Watery	Transparent
TUTVUGH3	Ghana	Nyankpala	39		Padi-tuya	5	2	Irregular	Watery	Transparent
TUTVUGH4	Ghana	Nyankpala	40		Padi-tuya	3	<1	Round	Watery	Translucent
TUTVUSA57	South Africa	Klipplaatdrift	41		Padi-tuya	7	1	Round	Cream	Opaque
TUTVUSA31	South Africa	Nelspruit	42	F	Omandaw	5	2	Oval	Watery	Transparent
TUTVUSA32	South Africa	Nelspruit	43		Padi-tuya	5	4	Oval	Watery	Transparent
TUTVUGH1	Ghana	Nyankpala	44		Apagbaala	1	2	Round	Watery	Translucent
TUTVUSA30	South Africa	Nelspruit	45		IT90K-277-2	6	2	Round	Watery	Translucent

Growth refers to the number of days taken for colonies to appear on yeast mannitol agar plates.

### Rep-PCR (BOX-PCR) fingerprints of cowpea isolates

**Figure 1:**
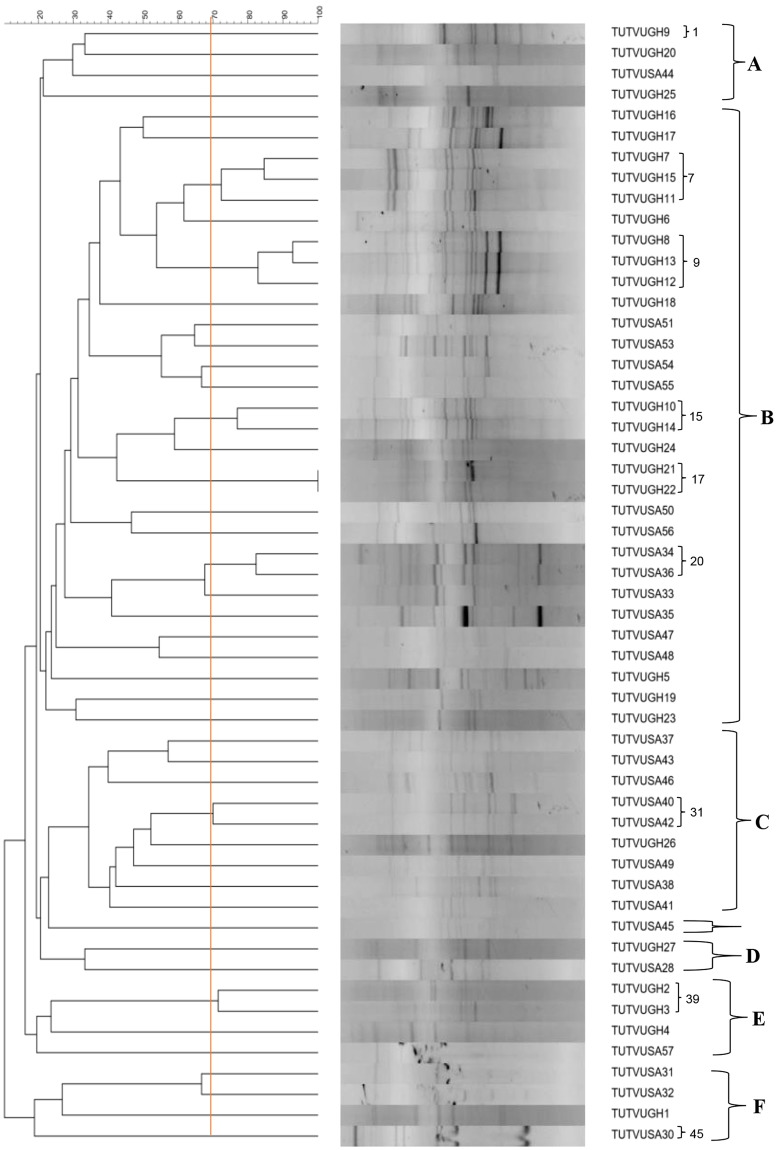
Genomic fingerprints of 54 cowpea microsymbionts from Ghana and South Africa. Bold alphabets indicate major clusters. Isolates having distinct Box-PCR profiles at a cut-off point of 70% similarity are indicated by means of Arabic numerals. Where consecutive isolates exhibit distinct PCR profiles, the numbering is skipped and continued at the next group of isolates. The PCR-amplified products were electrophoresed in 1.2% agarose gel (20 × 15 cm gel size) for 6 h at 85 volt. The sizes of bands were determined using the Image Lab software (Bio-Rad version 4.1). All bands were used for cluster analysis with the UPGMA (Unweighted Pair Group Method with Arithmetic mean) algorithm using the software Bionumerics 7.6. Gel images are supplied in the supplementary file.

Genomic fingerprinting using BOX- PCR yielded variable band sizes ranging from 618 bp to 5354 bp. The isolates were grouped into six major clusters (Cluster **A**–**F**) in the constructed dendrogram (Fig. 1). Out of the 54 test isolates, 38 possessed unique Box-PCR profiles if considered at a cut-off point of 70% similarity level. Cluster **A** contained four isolates, each obtained from nodules of a different cowpea genotype, and comprised only one isolate (TUTVUSA41) from Klipplaatdrift in South Africa. The remaining three isolates (namely, TUTVUGH19, TUTVUGH20 and TUTVUGH25) originated from Damongo and Garu in Ghana (Fig. 1). Cluster **B** was the largest, and contained 18 isolates from Ghana and 12 from South Africa. Out of the 18 isolates from Ghana, 11 originated from Damongo, six from Garu and only one from Nyankpala. The isolates from South Africa within cluster **B** comprised four from the middleveld (Klipplaatdrift) and eight from the lowveld (Nelspruit) (Table 1; Fig. 1). Of the nine isolates in cluster **C**, only one isolate (TUTVUGH26 from IT90K-277-2 in Garu) was from Ghana, while the remaining eight were from Klipplaatdrift, South Africa (Fig. 1). Isolates TUTVUGH27 and TUTVUSA28 from Ghana and South Africa, respectively, formed Cluster **D**, while isolate TUTVUSA45 stood alone between Clusters **C** and **D** (Fig. 1). However, isolates TUTVUGH2, TUTVUGH3 and TUTVUGH4 from Nyankpala, as well as TUTVUSA57 from South Africa, grouped together in Cluster **E**. Except for TUTVUGH1 which was isolated from root nodules of genotype Apagbaala from Nyankpala in Ghana, the other three isolates (TUTVUSA30, TUTVUSA31 and TUTVUSA32) in Cluster **F** were respectively obtained from genotypes IT90K-277-2, Omandaw and Padi-tuya, planted at Nelspruit, South Africa (Fig. 1; Table 1). There was a general tendency for isolates within each cluster to group in close proximity based on their locations of origin (Table 1). In this study, however, cowpea genotypes did not influence isolate groupings since most clusters contained isolates from different cowpea genotypes (Table 1).

### Phylogeny of cowpea isolates based on 16S rRNA analysis

**Figure 2:**
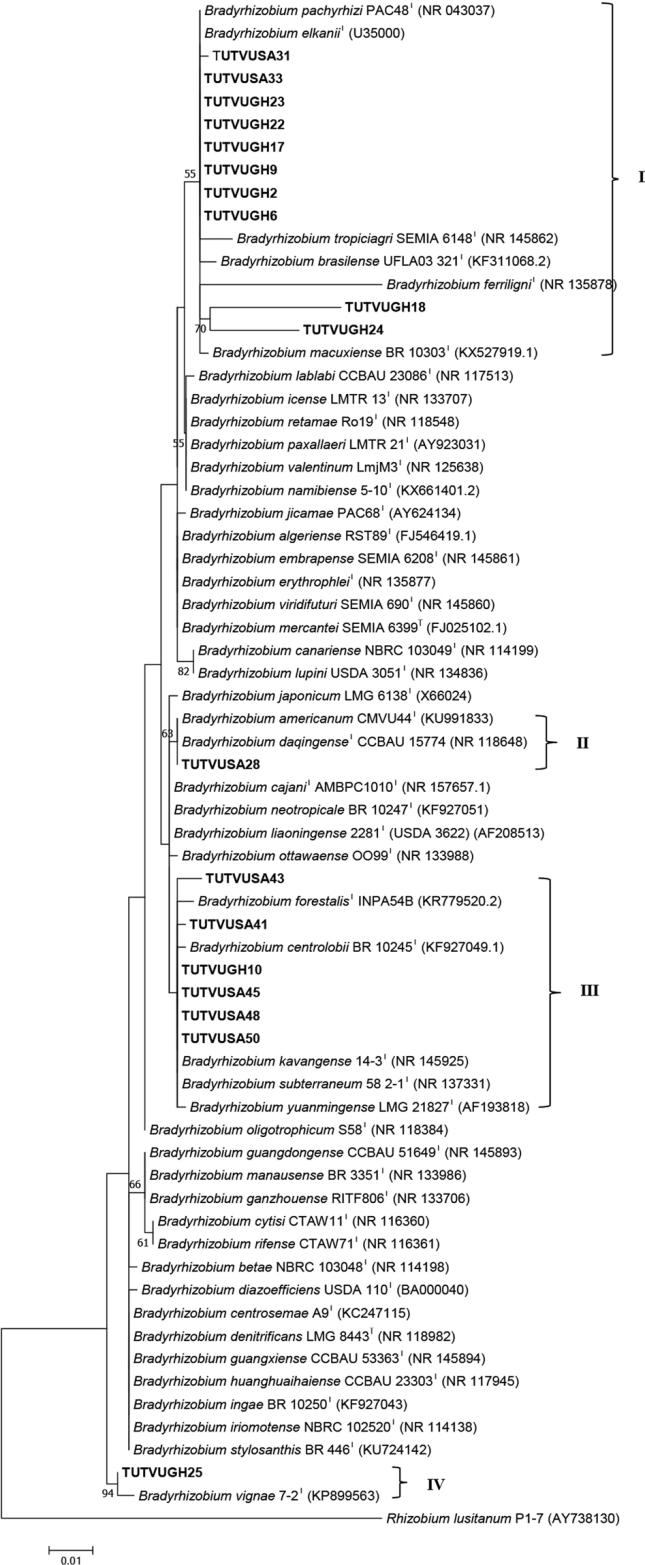
Maximum likelihood molecular phylogenetic analysis of cowpea nodulating rhizobia from Ghana and South Africa based on 16S rRNA gene sequences. The evolutionary history was inferred by using the Maximum Likelihood method based on the Kimura 2-parameter model^50^. The tree is drawn to scale, with branch lengths measured in the number of substitutions per site. The analysis involved 67 nucleotide sequences. All positions containing gaps and missing data were eliminated. Evolutionary analyses were conducted in MEGA7^49^.

The amplification of 16S rRNA region of bacterial genome yielded approximately 1500 bp length bands for each test isolate. Sequences of 16S rRNA gene obtained from isolates selected from each cluster of BOX-PCR showed 121 variables and 21 parsimony-informative regions (Table S1). The 16S rRNA gene-based phylogeny assigned all the selected isolates to the genus *Bradyrhizobium* (Fig. 2), and placed them in four groups (Group I - IV) within the phylogenetic tree. In Group I, isolates TUTVUGH2, TUTVUGH6, TUTVUGH9, TUTVUGH17, TUTVUGH22, and TUTVUGH23 obtained from Ghana and TUTVUSA31 and TUTVUSA33 from South Africa shared 99.8–100% sequence similarity and 55% bootstrap support with *B. elkanii* and *B. pachyrhizi*, while isolates TUTVUGH18 and TUTVUGH24 which shared 95.2% sequence similarity with each other, clustered together and formed an outgroup within Group I (Fig. 2). In contrast, isolate TUTVUSA28 clustered with *B. daqingense* and *B. americanum* in Group II with 100% sequence homology and 63% bootstrap support. In Group III, however, isolates TUTVUGH10 from Ghana and TUTVUSA41, TUTVUSA43, TUTVUSA45, TUTVUSA48 and TUTVUSA50 from South Africa grouped with *B. kavangense, B. subterraneum, B. yuanmingense* and *B. centrolobii* with 99.2–100% sequence identity. In Group IV, isolate TUTVUGH25 from Ghana formed a separate cluster with *B. vignae* and shared 99.4% sequence identity and 94% bootstrap support (Fig. 2).

### Phylogeny based on protein-coding genes (*atpD, glnII, gyrB* and *rpoB*)

PCR-amplification of *atpD, glnII, gyrB and rpoB* genes which are respectively responsible for coding ATP synthase subunit beta, glutamine synthetase II, DNA gyrase subunit B and RNA polymerase subunit B yielded 600 bp, 650 bp, 600 bp and 550 bp band lengths. The sequence analysis of these individual genes respectively revealed 153, 189, 350, and 148 variable and 57, 151, 173, and 91 parsimony-informative regions for *atpD*, *glnII*, *gyrB*, and *rpoB* (Table S1). Except for a few instances, the phylogenies based on individual housekeeping genes were consistent with each other and congruent with the 16S rRNA analysis (Fig. 2; Figs S1–S4). For example, although isolate TUTVUGH2 clustered with *B. elkanii* in the 16S rRNA and *glnII* phylograms with sequence identity of 100% and 97.6%, respectively, the same isolate stood alone in the *gyrB* gene phylogeny and clustered with *B. viridifuturi* in the *rpoB* phylogeny with 98.7% sequence identity (Fig. 2; Figs S1–S4).

### Concatenated sequence and phylogenetic analysis

**Figure 3:**
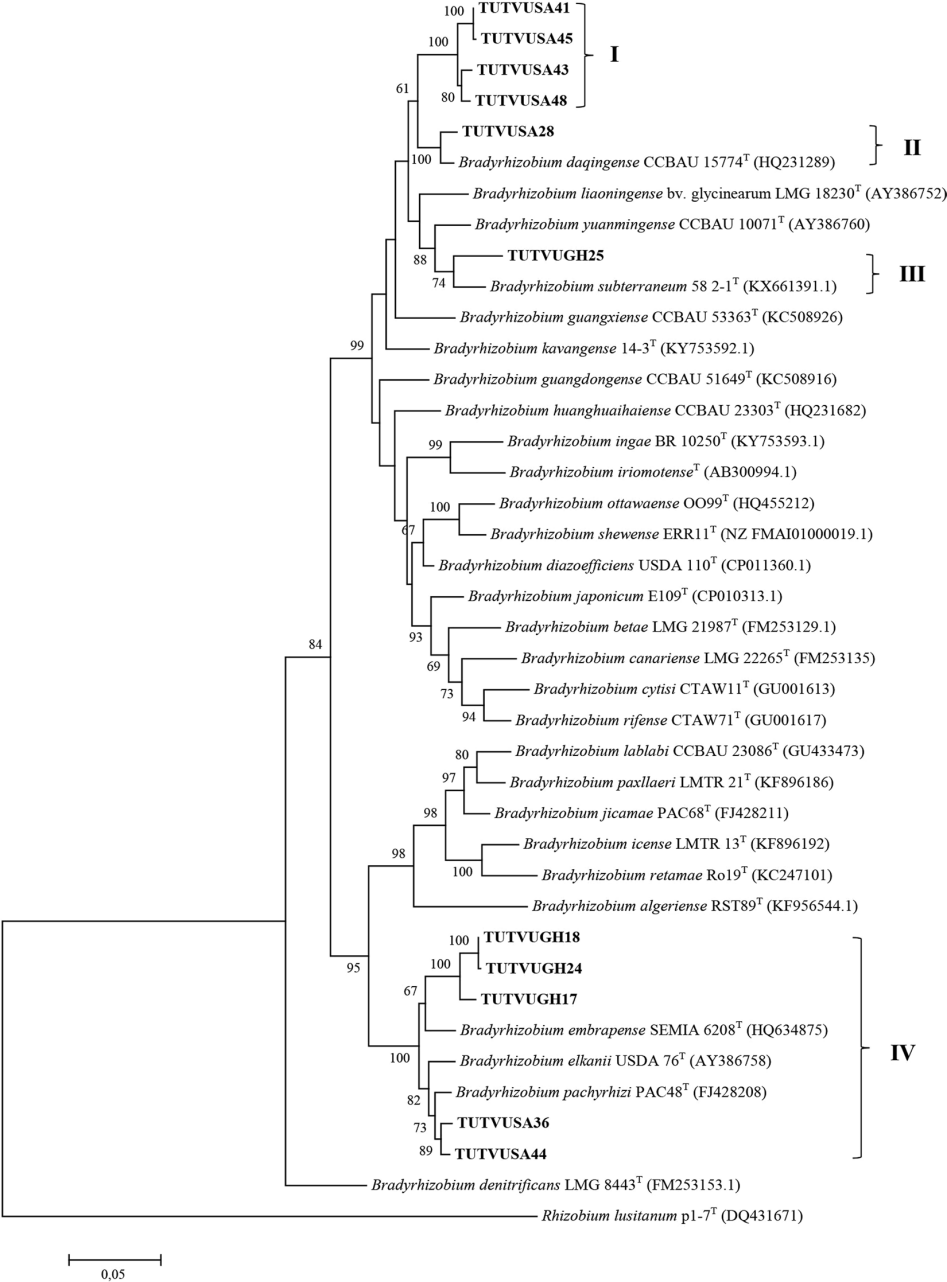
Maximum likelihood phylogeny of cowpea nodulating rhizobia from Ghana and South Africa based on concatenated sequences of *atpD*-*glnII*-*gyrB*-*rpoB* genes. The evolutionary history was inferred by using the Maximum Likelihood method based on the Kimura 2-parameter model^50^. The percentage of trees in which the associated taxa clustered together is shown next to the branches. The tree is drawn to scale, with branch lengths measured in the number of substitutions per site. The analysis involved 40 nucleotide sequences. We used only those strains which were present in all test gene phylogenies. Codon positions included were 1st + 2nd + 3rd + Noncoding. All positions containing gaps and missing data were eliminated. There were a total of 1523 positions in the final dataset. Evolutionary analyses were conducted in MEGA7^49^.

**Figure 4:**
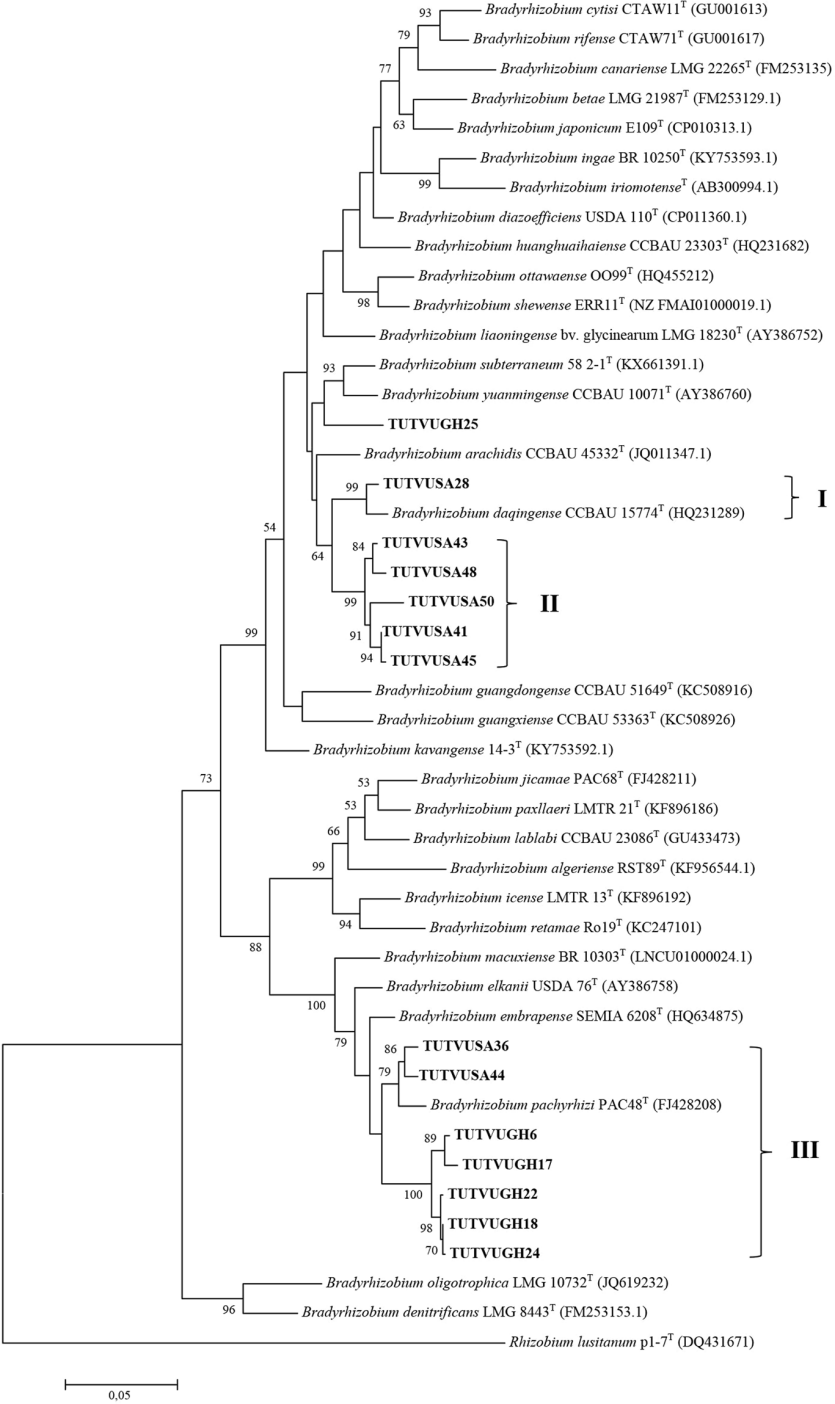
Maximum likelihood phylogeny of cowpea nodulating rhizobia from Ghana and South Africa based on concatenated sequences of *atpD*-*glnII*-*rpoB* genes. The evolutionary history was inferred by using the Maximum Likelihood method based on the Kimura 2-parameter model^50^. Bootstrap values >50% are given at the branching nodes. The percentage of 1000 replicates calculated under distance criteria in which the associated taxa clustered together is shown next to the branches. A discrete Gamma distribution was used to model evolutionary rate differences among sites. The tree is drawn to scale, with branch lengths measured in the number of substitutions per site. The analysis involved 46 nucleotide sequences. Codon positions included were 1st + 2nd + 3rd + Noncoding. All positions containing gaps and missing data were eliminated. There were a total of 1027 positions in the final dataset. Sequences were aligned using Clustal W and evolutionary analyses were conducted in MEGA6^49^.

Due to inconsistencies in the number of gene sequences obtained for the test isolates, two concatenated sequences and phylogenetic analyses were done. The variations in number of sequences obtained were due to failure to amplify the gene in some isolates, as well as to poor quality of sequences obtained in some instances. Two phylogenetic trees were constructed based on concatenated sequences of *atpD*-*glnII*-*gyrB*-*rpoB* genes for 11 isolates, and *atpD*-*glnII*-*rpoB* for 14 isolates (Figs 3 and 4). The phylograms obtained for concatenated sequences of four (Fig. 3) or three (Fig. 4) housekeeping genes were congruent to each other and to phylograms based on individual gene sequences. The phylogenies obtained from concatenated sequences of housekeeping genes placed cowpea isolates TUTVUSA41, TUTVUSA43, TUTVUSA45 and TUTVUSA48 together (Group I) in the *atpD*-*glnII*-*gyrB*-*rpoB* phylogram (100% bootstrap support and 98.3–99.8% sequence similarity), and also clustered them with isolate TUTVUSA50 in the *atpD*-*glnII*-*rpoB* phylogram (99% bootstrap support and 98.0–99.8% sequence identity; see Group II) but stood separately without any reference *Bradyrhizobium* strains (Figs 3 and 4). Although isolates TUTVUSA41, TUTVUSA43, TUTVUSA45, TUTVUSA48 and TUTVUSA50 showed closer relationship with *B. kavangense, B. subterraneum, B. yuanmingense* and *B. centrolobii* (99.2–100% sequence identity) in the 16S rRNA phylogeny (Fig. 2), these same isolates were distantly related to both type strains in the *atpD*, *glnII*, *gyrB* and *rpoB* phylogenies (Figs S1–S4). However, they shared sequence similarities of 95.1–95.2% with *B. daqingense* (the closest type strain) in the *atpD*-*glnII*-*gyrB*-*rpoB* (Figs. 3), and 94.5–95.7% with *B. daqingense* and *B. arachidis* in the *atpD*-*glnII*-*rpoB* concatenated phylogenies (Fig. 4). Furthermore, cowpea isolates TUTVUGH6, TUTVUGH17, TUTVUGH18, TUTVUGH22, TUTVUGH24 from Ghana and TUTVUSA36 and TUTVUSA44 from South Africa formed Group III in the *atpD*-*glnII*-*rpoB* phylogram. Within this cluster (Group III), the isolates from Ghana stood alone and shared 96.0–96.1% sequence similarity with *B. embrapense* SEMIA 6208^T^ with 100% bootstrap support while those from South Africa (i.e. TUTVUGH36 and TUTVUGH44) shared 97.9% sequence similarity with *B. pachyrhizi* PAC 48^T^ with 79% bootstrap support (Figs 3 and 4). Isolate TUTVUSA28 grouped with reference strain *B. daqingense* CCBAU 15774^T^ in the *atpD*-*glnII*-*gyrB*-*rpoB* and *atpD*-*glnII*-*rpoB* phylogenies, with sequence similarities of 98.3% and 98.5%, and bootstrap supports of 100% and 99%, respectively (Figs 3 and 4). Although isolate TUTVUGH25 grouped with *B. vignae* in the 16S rRNA, *glnII* and *rpoB* phylogenies with 99.4%, 99.3% and 95.3% sequence identities, respectively, the isolate also grouped with *B. subterraneum* in the *gyrB* phylogram and in the concatenated *atpD*-*glnII*-*gyrB*-*rpoB* phylogeny with 95.8% and 95.7% sequence similarities, respectively, due to the absence of *B. vignae* in these two phylograms.

### Phylogeny based on symbiotic (*nifH* and *nodC*) genes

**Figure 5:**
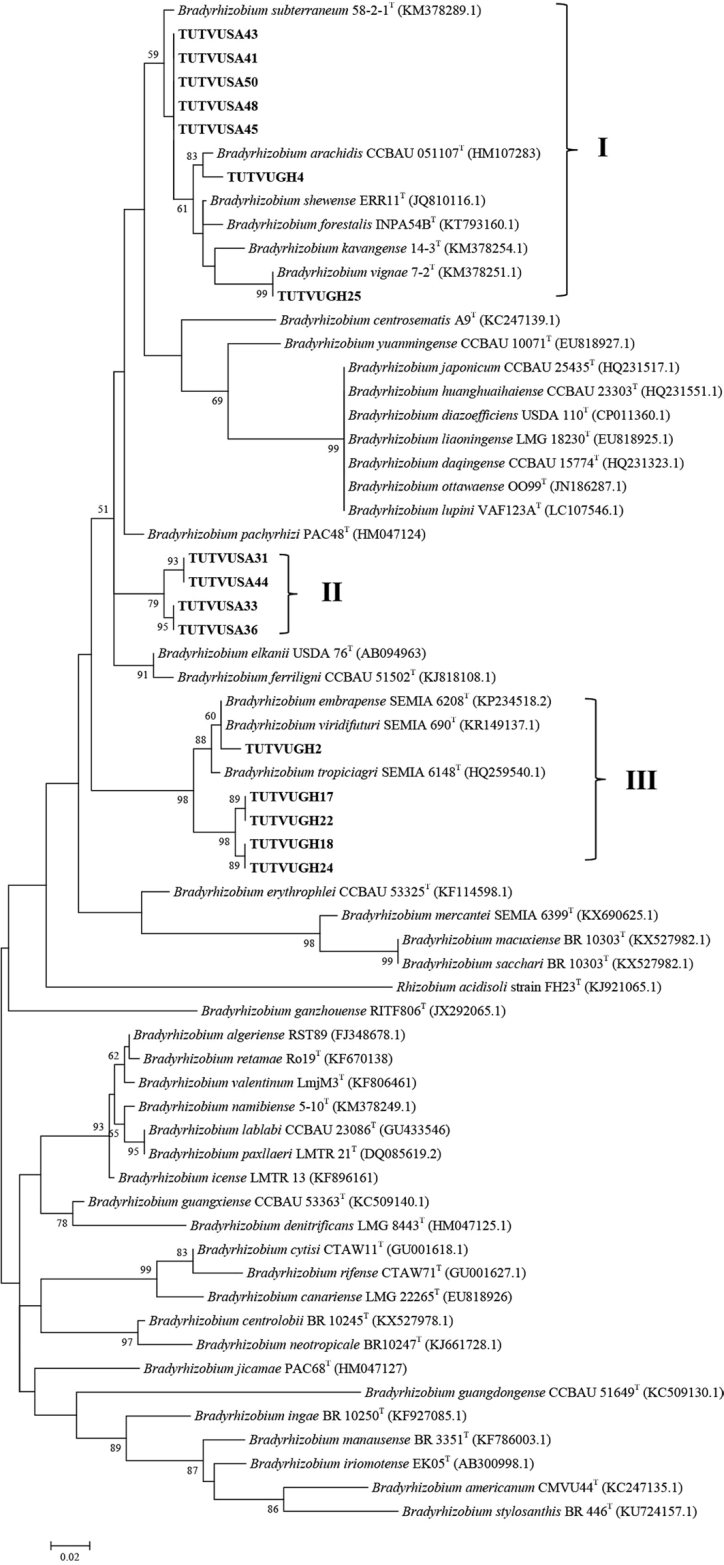
Maximum likelihood molecular phylogenetic analysis of cowpea nodulating rhizobia from Ghana and South Africa based on *nifH* gene sequences with type strains of *Bradyrhizobium* species. The evolutionary history was inferred by using the Maximum Likelihood method based on the Kimura 2-parameter model^50^. The scale bar indicates the number of substitutions per site. The percentage of trees in which the associated taxa clustered together is shown next to the branches. The analysis involved 64 nucleotide sequences. Codon positions included were 1st + 2nd + 3rd + Noncoding. All positions containing gaps and missing data were eliminated. Evolutionary analyses were conducted in MEGA7^49^.

**Figure 6:**
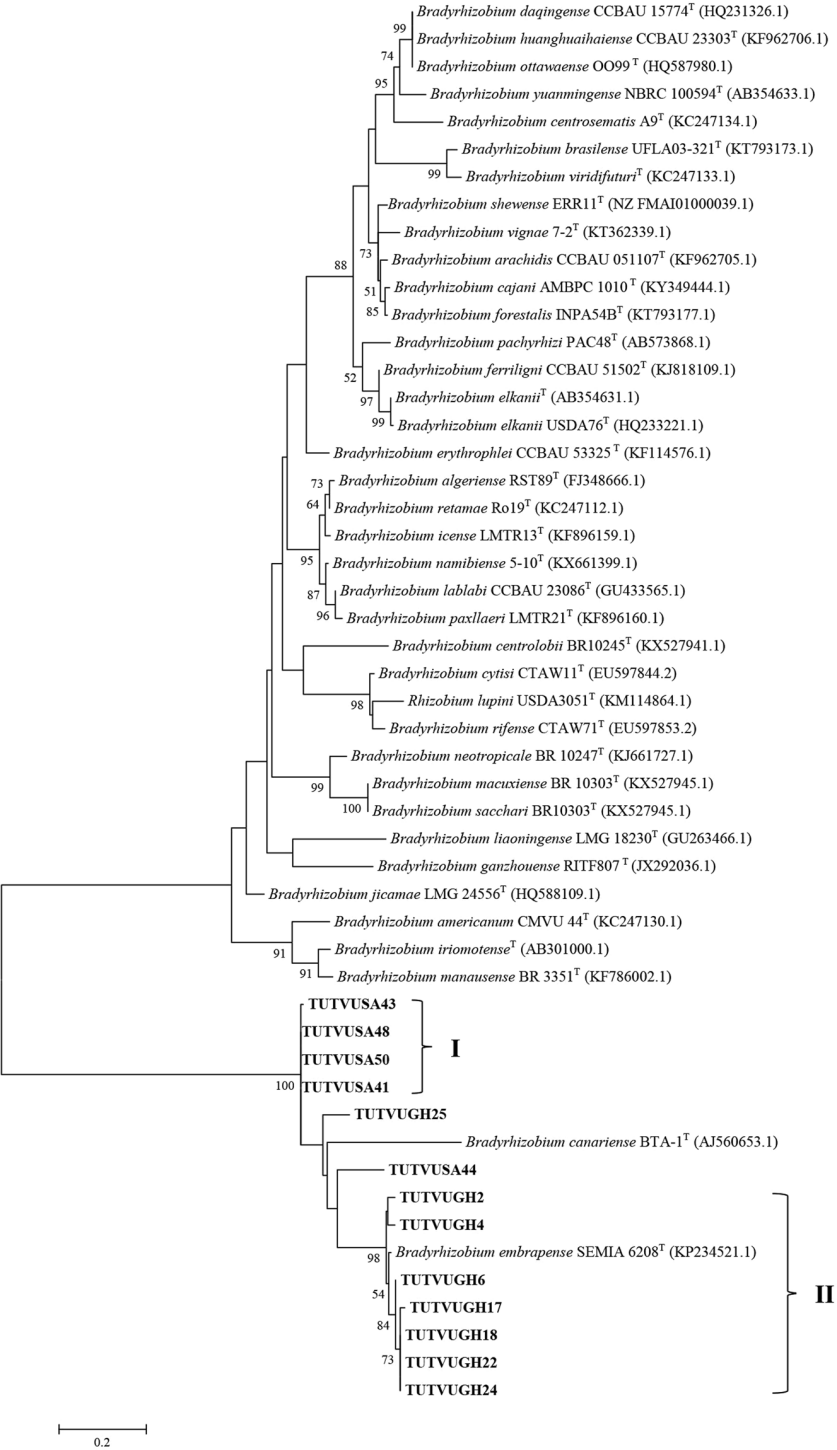
Maximum likelihood molecular phylogenetic analysis of cowpea nodulating rhizobia from Ghana and South Africa with type *Bradyrhizobium* strains based on *nodC* gene sequences. The evolutionary history was inferred by using the Maximum Likelihood method based on the Kimura 2-parameter model^50^. The percentage of trees in which the associated taxa clustered together is shown next to the branches. The tree is drawn to scale, with branch lengths measured in the number of substitutions per site. The analysis involved 51 nucleotide sequences. Codon positions included were 1st + 2nd + 3rd + Noncoding. All positions containing gaps and missing data were eliminated. Evolutionary analyses were conducted in MEGA7^49^.

Phylogenetic analysis of *nifH* and *nodC* genes assigned all the test cowpea isolates to distinct clusters within the *Bradyrhizobium* genus (Figs 5 and 6). The phylogenies from analysis of both symbiotic genes (*nifH* and *nodC*) were congruent with individual housekeeping genes, and with phylogenies based on concatenated gene sequences. Isolates TUTVUSA41, TUTVUSA43, TUTVUSA45, TUTVUSA48 and TUTVUSA50 clustered together and shared 99.0% sequence homology with *B. subterraneum* 58-2-1^T^ and 59% bootstrap support in Group I of the *nifH* phylogeny (Fig. 5). These same isolates (except for TUTVUSA45 which showed poor sequence analysis), again grouped together in the *nodC* phylogeny with 99.4–100% sequence similarity and 100% bootstrap support (Fig. 6). Within the same Group I in the *nifH* phylogram, isolates TUTVUGH4 and TUTVUGH25 were separately grouped, and showed close relationship with *B. arachidis* and *B. vignae* with 98.5% and 100% sequence similarities respectively with the type strains. Isolates TUTVUSA31, TUTVUSA33, TUTVUSA36 and TUTVUSA44 also clustered together and stood separately from the reference type strains in the *nifH* phylogram but shared 94.5–95.5% sequence similarity with *B. pachyrhizi* PAC 48^T^ and *B. shewense* ERR11^T^ in Group II (Fig. 5). Furthermore, isolates TUTVUGH17, TUTVUGH18, TUTVUGH22 and TUTVUGH24 clustered together in Group III of the *nifH* phylogeny and shared 96.5% sequence similarity with *B. tropiciagri* with 98% bootstrap support (Fig. 5). But within the same Group III, isolate TUTVUGH2 showed a distinctly closer relationship with *B. embrapense* SEMIA 6208^T^ and *B. viridifuturi* SEMIA 690^T^ with 99% sequence similarity and 60% bootstrap support (Fig. 5). With the *nodC* phylogeny, however, isolates TUTVUGH2, TUTVUGH4 as well as TUTVUGH6, TUTVUGH17, TUTVUGH18, TUTVUGH22 and TUTVUGH24 grouped together (Group I) and shared 96.8–97.9% sequence similarity with *Bradyrhizobium embrapense* SEMIA 6208^T^ belonging to the symbiovar *tropici*, the closest reference type strain (Fig. 6). On the other hand, isolates TUTVUGH25 and TUTVUSA44 formed outgroups of isolates in Group II in the *nodC* phylogeny, but respectively shared 80.7% and 79.6% sequence homology with *B. embrapense* SEMIA 6208^T^, the closest type strain (Fig. 6).

### Gas-exchange parameters/symbiotic effectiveness of nodule isolates

Inoculating cowpea with the test isolates resulted in variable (p ≤ 0.001) photosynthetic rates, leaf transpiration and total chlorophyll. The commercial *Bradyrhizobium* strain CB756 induced the highest photosynthetic rates (22.3 µmol ms^−2^s^−1^), followed by isolate TUTVUSA28 (21.5 µmol ms^−2^s^−1^), and TUTVUGH18 (20.8 µmol ms^−2^s^−1^). All test isolates, including the commercial *Bradyrhizobium* strain CB756 elicited greater total leaf chlorophyll and photosynthesis when compared to the 5 mM KNO_3_-fed plants. In general, increased photosynthetic rates were mostly associated with higher leaf transpiration, stomatal conductance and greater chlorophyll in leaves (Table 2).

**Table 2:** Nodulation (nodule number and nodule dry matter), shoot dry matter, photosynthesis (A), stomatal conductance (gs), transpiration (E), leaf total chlorophyll (Total Chl.) and relative effectiveness (RE) induced by indigenous rhizobial isolates of cowpea relative to inoculation with *Bradyrhizobium* strain CB756 or 5 mM KNO_3_ feeding.

Treatment	Nodule No.	Nodule DM	Shoot DM	A	gs	E	Total Chl.	RE
	plant^−1^	mg.plant^−1^	g. plant^−1^	µmol (CO_2_) ms^−2^s^−1^	mol (H_2_O) ms^−2^s^−1^	mol (H_2_O) ms^−2^s^−1^	(mg. g^−1^. Fresh wt.)	%
TUTVUGH2	59 ± 5.4c-f	65.1 ± 10.3 h	1.01 ± 0.09k	16.7 ± 1.01gh	0.31 ± 0.04b-f	7.1 ± 0.48def	1.30 ± 0.06gh	45 ± 3.8k
TUTVUGH4	47 ± 4.9e-h	80.1 ± 10.5gh	1.71 ± 0.27hij	19.7 ± 0.82b-e	0.43 ± 0.00a	9.1 ± 0.02a	1.34 ± 0.14gh	76 ± 12.1hij
TUTVUGH17	65 ± 8.2b-e	120.2 ± 8.1c-f	2.21 ± 0.49fgh	18.6 ± 1.04d-g	0.30 ± 0.02c-f	7.8 ± 0.17cde	1.56 ± 0.15fg	99 ± 21.7fgh
TUTVUGH18	74 ± 4.3bcd	154.4 ± 10.5abc	3.32 ± 0.05abc	20.8 ± 0.92abc	0.43 ± 0.00a	8.9 ± 0.00abc	2.21 ± 0.08abc	148 ± 2.4abc
TUTVUGH22	53 ± 7.0d-g	108.7 ± 19.1d-g	2.55 ± 0.11def	16.3 ± 0.00 h	0.19 ± 0.00 hi	5.7 ± 0.00h	1.89 ± 0.01cde	114 ± 5.1def
TUTVUGH24	62 ± 8.7cde	89.5 ± 16.0fgh	1.74 ± 0.31hij	19.9 ± 0.35bcd	0.39 ± 0.04ab	8.4 ± 0.38a-d	1.47 ± 0.14fg	78 ± 13.6hij
TUTVUGH25	66 ± 2.1b-e	92.2 ± 17.0e-h	2.35 ± 0.04efg	18.2 ± 0.01d-h	0.22 ± 0.03gh	6.3 ± 0.59gh	2.42 ± 0.16a	105 ± 1.9efg
TUTVUSA28	34 ± 2.5fgh	128.2 ± 2.3c-e	2.37 ± 0.05efg	21.5 ± 0.60ab	0.30 ± 0.02c-f	8.8 ± 0.30abc	1.46 ± 0.05fg	106 ± 2.2efg
TUTVUSA31	35 ± 6.3fgh	70.9 ± 7.4gh	1.17 ± 0.07jk	18.4 ± 0.79d-g	0.33 ± 0.04bcd	8.0 ± 0.81bcd	1.11 ± 0.06 h	52 ± 3.0jk
TUTVUSA33	83 ± 8.2bc	159.7 ± 9.0ab	3.10 ± 0.29bcd	20.1 ± 0.01bcd	0.26 ± 0.00e-h	8.6 ± 0.01a-d	2.06 ± 0.02bc	138 ± 13.0bcd
TUTVUSA36	75 ± 5.0bcd	105.3 ± 12.2d-h	2.81 ± 0.30c-f	19.0 ± 1.41c-f	0.33 ± 0.02b-e	9.0 ± 0.38ab	2.27 ± 0.04ab	125 ± 13.5c-f
TUTVUSA41	125 ± 12.1a	143.3 ± 3.4a-d	3.71 ± 0.04a	16.2 ± 0.01 h	0.25 ± 0.03fgh	5.9 ± 0.47gh	2.02 ± 0.01bcd	166 ± 1.7a
TUTVUSA43	23 ± 4.9 h	86.9 ± 25.2fgh	2.44 ± 0.01ef	12.4 ± 0.00i	0.15 ± 0.00i	4.3 ± 0.02i	1.68 ± 0.10ef	109 ± 0.5ef
TUTVUSA44	88 ± 0.8b	136.5 ± 3.8a-d	2.89 ± 0.11cde	19.6 ± 0.45b-e	0.32 ± 0.02b-e	6.4 ± 0.21gh	2.29 ± 0.08ab	129 ± 5.1cde
TUTVUSA45	33 ± 3.7gh	74.9 ± 4.9gh	1.81 ± 0.09ghi	18.2 ± 0.47d-h	0.35 ± 0.04bc	6.8 ± 0.47efg	2.01 ± 0.05bcd	81 ± 4.2ghi
TUTVUSA48	74 ± 16.3bcd	74.6 ± 11.9gh	1.49 ± 0.07ijk	17.7 ± 0.01e-h	0.34 ± 0.01bc	6.6 ± 0.15fgh	2.08 ± 0.09bc	66 ± 3.1ijk
TUTVUSA50	114 ± 14.0a	170.0 ± 3.6a	3.57 ± 0.20ab	17.1 ± 0.09fgh	0.26 ± 0.01d-g	6.9 ± 0.05efg	1.71 ± 0.10def	159 ± 9.0ab
*Bradyrhizobium* CB756	59 ± 11.7c-g	72.2 ± 8.1gh	2.78 ± 0.17c-f	22.3 ± 0.00a	0.35 ± 0.00bc	6.5 ± 0.00fgh	1.97 ± 0.19b-e	124 ± 7.8c-f
5 mM KNO_3_	NA	NA	2.24 ± 0.03fgh	7.0 ± 0.10j	0.07 ± 0.00j	2.5 ± 0.05j	0.58 ± 0.04i	NA
**F statistics**	11.2***	8.02***	16.2***	31.5***	16.2***	25.2***	22.0***	16.4***
**cv (%)**	**24.6**	**22.5**	**16**	**6.9**	**15.5**	**9.7**	**11.4**	**16.3**

Values (means ± SE; n = 4) with dissimilar letters in a column are significantly different at p < 0.001 (***).

NA = Not applicable; RE was calculated as (x/y) * 100 for only the inoculated treatments. Where: x = shoot DM of inoculated plants and y = shoot DM of 5 mM KNO_3_-fed plants.

The test isolates induced variable (p ≤ 0.001) nodule number and nodule dry matter on cowpea plants (Table 2). Isolates TUTVUSA41 and TUTVUSA50 elicited the highest nodule formation on cowpea (the homologous host), while TUTVUSA43 and TUTVUSA45 induced the least nodule number (Table 2) even though the four isolates shared the same cluster in all phylogenetic trees. In fact, high shoot dry weights were also recorded by isolates TUTVUSA41 and TUTVUSA50, which exhibited superior symbiotic efficiency with relative effectiveness values of 166% and 159%, respectively (Table 2).

### Soil influence on bradyrhizobial distribution

**Figure 7:**
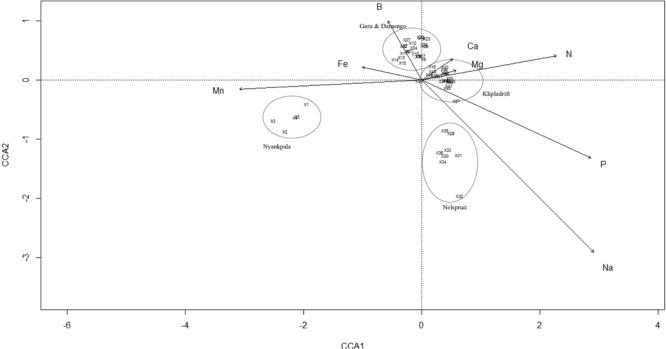
Canonical correspondence analysis (CCA) showing the influence of soil chemical properties on the distribution of cowpea nodulating rhizobia from different locations in Ghana and South Africa. Names of locations in CCA biplot are accessory variables and were not included in the analysis. In the plot, the isolates are numbered as: X1 = TUTVUGH1, X2 = TUTVUGH2, X3 = TUTVUGH3, X4 = TUTVUGH4, X5 = TUTVUGH5, X6 = TUTVUGH6, X7 = TUTVUGH7, X8 = TUTVUGH8, X9 = TUTVUGH9, X10 = TUTVUGH10, X11 = TUTVUGH11, X12 = TUTVUGH12, X13 = TUTVUGH13, X14 = TUTVUGH14, X15 = TUTVUGH15, X16 = TUTVUGH16, X17 = TUTVUGH17, X18 = TUTVUGH18, X19 = TUTVUGH19, X20 = TUTVUGH20, X21 = TUTVUGH21, X22 = TUTVUGH22, X23 = TUTVUGH23, X24 = TUTVUGH24, X25 = TUTVUGH25, X26 = TUTVUGH26, X27 = TUTVUGH27, X28 = TUTVUSA28, X30 = TUTVUSA30, X31 = TUTVUSA31, X32 = TUTVUSA32, X33 = TUTVUSA33, X34 = TUTVUSA34, X35 = TUTVUSA35, X36 = TUTVUSA36, X37 = TUTVUSA37, X38 = TUTVUSA38, X40 = TUTVUSA40, X41 = TUTVUSA41, X42 = TUTVUSA42, X43 = TUTVUSA43, X44 = TUTVUSA44, X45 = TUTVUSA45, X46 = TUTVUSA46, X47 = TUTVUSA47, X48 = TUTVUSA48, X49 = TUTVUSA49, X50 = TUTVUSA50, X51 = TUTVUSA51, X53 = TUTVUSA53, X54 = TUTVUSA54, X55 = TUTVUSA55, X56 = TUTVUSA56, X57 = TUTVUSA57.

CCA analysis was used to correlate bradyrhizobial distribution with environmental variables such as the concentrations of micro- and macronutrients (namely, B, Fe, Mn, Ca, Mg, Na, N, P, K, and soil pH) in soils (Fig. 7). A CCA ordination graph was constructed with only those variables which showed significant influence on bradyrhizobial distribution (p < 0.001 at permutation 999). In the CCA plot, the total mean square contingency coefficient (inertia) was 13.16, of which 10% was constrained (explainable) and 90% was unconstrained (unexplainable). Only 6.2% of the variation was explained by both CCA1 and CCA2 axes. As shown in the CCA plot, the concentrations of Mn, Fe and N in soils showed stronger correlations with the first canonical axis (CCA1), although the effect of N was in the opposite direction (Fig. 7). Similarly, soil B and Na showed significant correlations with the second canonical axis (CCA2), although in opposite directions. Nevertheless, both B and Na also contributed to determine the first canonical axis but to a lower extent compared to Mn, N and Fe (Fig. 7). On the other hand, the concentrations of Mg and Ca in soils showed slight correlations with the CCA1 axis. However, soil Ca and Na contents were orthogonal to each other (Fig. 7). The CCA plot revealed that, the concentration of N, Mg, Ca, Mn and Fe exerted a stronger influence on the genomic variations among the bradyrhizobial isolates with the CCA1, followed by B, P and Na with the CCA2 axis. Among the test locations, the isolates from Nyankpala in Ghana were closely related to soil Mn. On the other hand, the isolates obtained from Kliplaadrift in South Africa were highly influenced by increasing soil N, Mg, Ca and P while those from Nelspruit were influenced by the concentration of Na. Furthermore, the concentration of B in soils showed a strong influence on the isolates obtained from Garu and Damongo both in Ghana (Fig. 7).

### Phylogenetic and functional correlations

**Figure 8:**
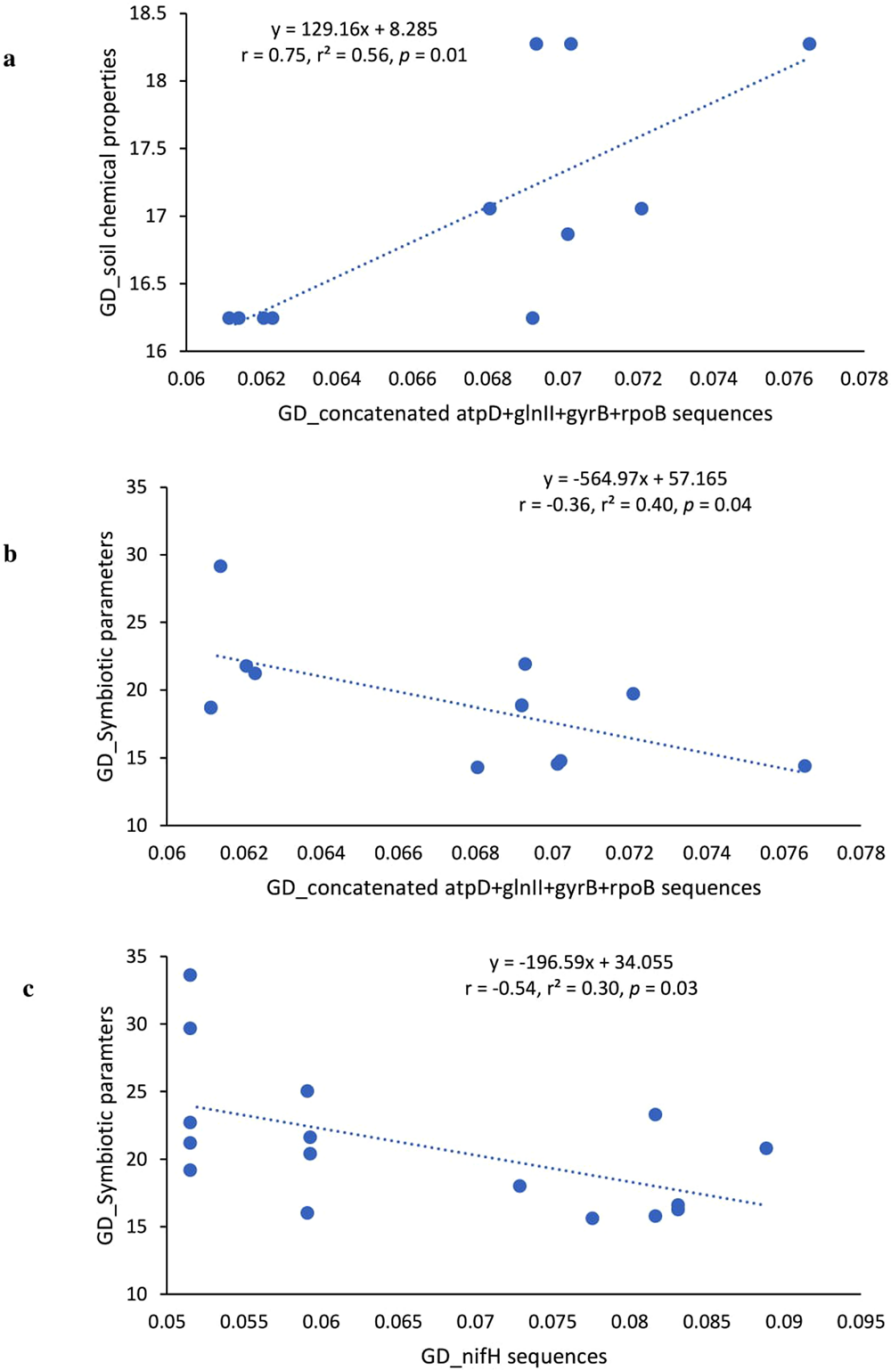
Correlation and regression analysis between genetic distances (GD) of isolates based on (**a**) soil chemical properties and concatenated sequences of *atpD* + *glnII* + *gyrB* + *rpoB*, (**b**) symbiotic parameters and concatenated sequences of *atpD* + *glnII* + *gyrB* + *rpoB* and (**c**) symbiotic parameters and *nifH* gene sequences. The genetic distances based on gene sequences were generated using Kimura 2-parameter matrices in MEGA7 software^49^, while the genetic distances based on soil chemical properties and symbiotic parameters were generated using NTSYSpc software, version 2.21^52^.

The correlations performed between genetic distances obtained from the phylogenies of concatenated housekeeping genes and *nifH* genes and soil chemical properties or symbiotic parameters were significant (p < 0.05). The results showed that concatenated housekeeping gene phylogeny and soil chemical properties were highly correlated (r = 0.75, *p = *0.01) (Fig. 8a). The relationship between genetic distances of both concatenated housekeeping genes and *nifH* gene phylogenies versus the genetic distance of symbiotic parameters was assessed and found to be significant (r = −0.36, *p = *0.04; r = −0.54, *p* = 0.03, respectively), though negatively (Fig. 8b,c).

## Discussion

**Figure 9:**
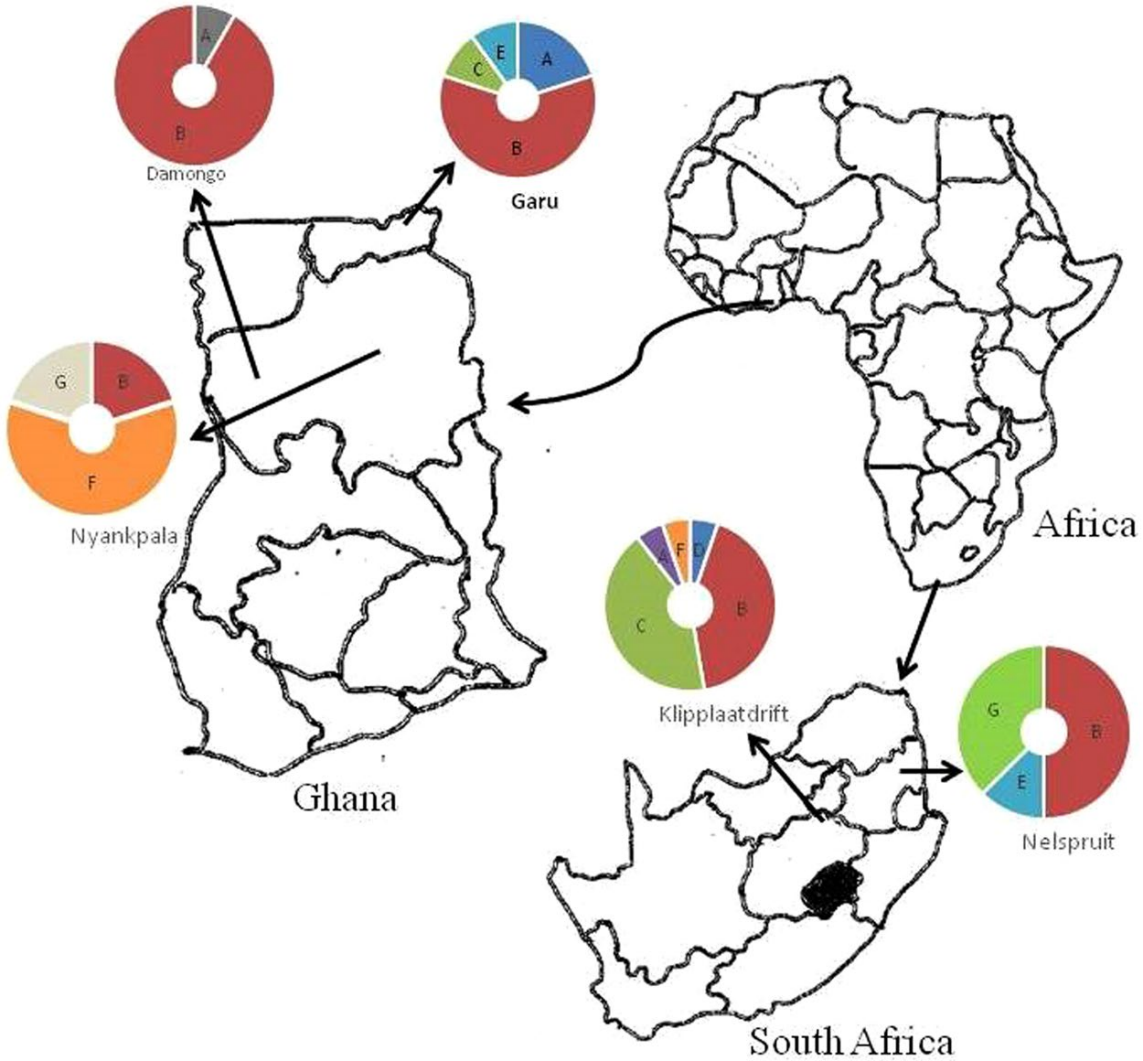
African test locations and rhizobial occupation in different Box-PCR clusters. For each location, the number of segments indicate the number of Box-PCR clusters occupied by isolates from that site. Uppercase letters in segments represent the labels of Box-PCR clusters (see Table 1). The area of each segment is proportional to the number of isolates from a given location occupying that cluster.

The isolation and subsequent symbiotic characterization of naturally adapted rhizobial symbionts of legumes constitute the first step to strain identification for inoculant production^20^. This study assessed the genetic diversity and phylogeny of rhizobial symbionts of cowpea from different locations in Ghana and South Africa, using rep-PCR (Box-PCR) fingerprinting and multilocus sequence analysis. The Box-PCR profiles revealed high genetic diversity among the native bacterial symbionts nodulating cowpea in its region of origin in Africa (Fig. 1). Isolates from Kliplaadrift in South Africa had the most diverse population, and occupied five major clusters (Figs 1 and 9). These results support a previous report which showed high genetic variation among bacterial symbionts nodulating cowpea in Botswana, Ghana and South Africa, with South African soils harbouring more diverse rhizobial populations^21^. Except for isolate TUTVUSA45 which stood alone in the dendrogram (Fig. 1), the remaining six major clusters identified in this study contained isolates from different locations in the two countries studied (Fig. 1; Table 1). There was however a general tendency for isolates within a cluster to group closely to each other based on their country of origin. The observed geographic distribution of the isolates in this study has been reported for cowpea-nodulating microsymbionts in Senegal, Greece and Mozambique^10,13,23^. Although the cowpea genotypes had no marked effect on the clustering of rhizobial isolates in this study (Table 1), they nevertheless influenced the diversity of nodule occupants. And this could be due to differences in the profile of *nod* gene-inducers present in seed and root exudates which differentially shaped the rhizobial microbiome in the rhizosphere and hence nodule occupancy^31^.

To infer the phylogenetic positions of cowpea isolates, 16S rRNA, protein-coding housekeeping (*atpD*, *glnII*, *gyrB* and *rpoB*) and symbiotic (*nifH* and *nodC*) genes were sequenced and analysed. As found in other studies of microsymbionts nodulating cowpea in Africa^10,13,28^, the isolates in this study clustered with *B. elkanii, B. daqingense*, *B. kavangense, B. subterraneum, B. yuanmingense* and *B. vignae* from sequence analysis of the 16S rRNA gene (Fig. 2). Despite the success of Box-PCR fingerprinting in determining genetic differences among cowpea isolates in this study, the approach was incongruent with the 16S rRNA-based phylogeny, a finding reported by Menna *et al*.^32^. However, the topologies of the phylograms constructed from the concatenated sequences of *atpD*-*glnII*-*gyrB*-*rpoB* and *atpD*-*glnII*-*rpoB* genes were consistent, and congruent with the 16S rRNA-based phylogram, thus allowing for a clearer delineation of the phylogenetic positions of the isolates used in this study. For example, isolates TUTVUSA41, TUTVUSA43, TUTVUSA45 and TUTVUSA48 grouped together in the single *atpD*, *glnII*, *gyrB* and *rpoB* gene phylogenies (Figs S1–S4). Isolates TUTVUGH17, TUTVUGH18, TUTVUGH24 from Ghana as well as TUTVUSA36 and TUTVUSA44 from South Africa similarly formed separate clusters within a major cluster in all the single gene phylogenies, as well as in the concatenated phylograms (Figs 3 and 4; Figs S1–S4). Isolates TUTVUSA41, TUTVUSA43, TUTVUSA45 and TUTVUSA48 from South Africa were tightly grouped in the two concatenated trees (Figs 3 and 4), and away from any reference type strains, despite being closer to *B. kavangense, B. subterraneum, B. centrolobii* and *B. yuanmingense* with 99.2–100% sequence identity in the 16S rRNA phylogeny. Although this could suggest that they are novel strains, a recent report by Helene *et al*.^33^ showed that high 16S rRNA sequence identity *per se* does not confirm isolate delineation with reference type strains. But isolates TUTVUSA36 and TUTVUSA44 were closely related to *B. pachyrhizi* PAC48^T^ (which was originally isolated from *Pachyrhizus erosus* in Costa Rica) with 97.9–98.3% sequence identity in the concatenated gene phylogenies^34^. These findings are consistent with previous reports which showed that *B. pachyrhizi* is associated with cowpea nodulation in the soils of Angola and Mozambique^13,20^. Isolates TUTVUGH17, TUTVUGH18, TUTVUGH24, TUTVUGH22 and TUTVUGH6 (all from Ghana) clustered with the *B. elkanii* group in the 16S rRNA phylogeny, but not closely with any known species in the other phylograms, suggesting that they could be novel species of *Bradyrhizobium*. Furthermore, our results also showed that isolate TUTVUSA28 consistently clustered in all phylogenies with *B. daqingense*, a strain originally isolated from soybean nodules in China and subsequently found to induce effective nodulation on cowpea^35^. The presence of *B. daqingense* in African soils could be caused by its introduction with soybean seeds from China, the country of origin of the crop^36^. But TUTVUGH25 was closely related to *B. vignae* which was initially isolated from cowpea in Namibia^37^. Interestingly, isolates TUTVUSA41, TUTVUSA43, TUTVUSA45, TUTVUSA48 in the concatenated *atpD*-*glnII*-*gyrB*-*rpoB* phylogeny, together with TUTVUSA50 in the *atpD*-*glnII*-*rpoB* phylogeny (see Group III) could be novel cowpea-nodulating symbionts since they shared low sequence homology (94.5–95.7%) with their closest related reference type strains in those phylograms (Figs 3 and 4).

The *nifH* and *nodC* phylogenies of cowpea isolates in this study were consistent with each other, and congruent with the 16S rRNA-based phylogeny which suggests coevolution of these symbiotic genes. Isolates TUTVUGH17, TUTVUGH18, TUTVUGH22 and TUTVUGH24 which shared 98.9–100% sequence identity in the symbiotic gene phylogenies grouped together and stood alone, but shared 96.5–97.5% sequence homology with *B. tropiciagri* and *B. embrapense*, which is an indication that they might represent novel species of the symbiovar *tropici*. Nevertheless, cowpea is a promiscuous host, and was recently reported to nodulate with different symbiovars in Africa and Europe^24^. Furthermore, the congruency of isolates TUTVUSA41, TUTVUSA43, TUTVUSA48 and TUTVUSA50 from South Africa and isolates TUTVUGH17, TUTVUGH18, TUTVUGH22 and TUTVUGH24 from Ghana in all the phylogenetic trees could suggest the maintenance of symbiotic genes through vertical gene transfer. Also, the close relationship of isolates TUTVUSA33 and TUTVUSA36 with *B. pachyrhizi* and the presence of many novel *Bradyrhizobium* species could be due to the unique edapho-climatic conditions of the study sites in Africa. Species of *B. pachyrhizi* bacteria had previously been isolated from cowpea in Mexico and Greece where the climates are warm and the soils acidic^23,38^, as found at the study sites in Africa. A previous study demonstrated the effect of soil factors on the survival, persistence and diversity of rhizobia in soils^39^, which in turn influenced plant nodulation and symbiotic N_2_ fixation^40^. In this study, soil mineral nutrients were found to influence the diversity of bradyrhizobial populations in South African and Ghanaian soils. For example, the South African isolates were highly influenced by the concentrations of N, P and Na in the soil. In the phylogenetic tree, those isolates (i.e. TUTVUSA41, TUTVUSA43, TUTVUSA45, TUTVUSA48 and TUTVUSA50) from South Africa were also found to be closely related to *B. subterraneum, B. kavangense, B. centrolobii* and *B. yuanmingense*. However, in China, the distribution of *B. yuanmingense* was found to be affected by soil K, although other *Bradyrhizobium* species were also associated with soils with high P concentration^41^. Han *et al*.^42^ also showed that rhizobial diversity was altered by soil phosphorus. In this study, the distribution of isolates from Ghana were strongly influenced by high soil B, Fe and Mn, suggesting the possible roles of these elements in the survival and functioning of microsymbionts in the Ghanaian environment.

Glasshouse evaluation of the cowpea isolates studied revealed marked differences in their symbiotic effectiveness as evidenced by the significantly large variations in plant nodulation, shoot biomass, leaf chlorophyll and photosynthetic rates induced by the bacterial isolates (Table 2). Cowpea nodulated by the test isolates showed markedly higher leaf chlorophyll which probably stimulated greater photosynthesis, stomatal conductance and transpiration rates when compared to the 5 mM KNO_3_-fed plants. The C sink strength of the nodulated cowpea plants induced greater photosynthetic rates which in turn increased biomass accumulation and promoted plant growth relative to nitrate-feeding^16^. The nitrate-fed plants recorded much lower chlorophyll content and leaf photosynthesis despite having similar shoot biomass as some inoculated plants, suggesting that N_2_ fixation stimulated greater photosynthesis than nitrate-feeding. A recent report by Kaschuk *et al*.^43^ also found that inoculating soybean plants with effective *Bradyrhizobium* strains maintained leaf chlorophyll content, stimulated greater photosynthetic rates and delayed senescence over nitrate-fed plants. In this study, effective N_2_ fixation in the nodulated cowpea plants therefore provided the needed N for chlorophyll and Rubisco biosynthesis, which resulted in enhanced photosynthetic rates^43^.

Furthermore, a comparison of the phylogenetic and functional genetic distances between test isolates showed a clearly significant (p < 0.05) relationship. The positive correlation found between concatenated gene phylogeny and soil properties could be due to niche conservation and mineral nutrition of the test isolates. The housekeeping genes used in this study are conserved in the isolates, which suggest that the phylogenetically-clustered isolates were similar in their environmental distribution. A significant but negative correlation was found when symbiotic parameters were plotted against *nifH* and concatenate genes phylogenies (Fig. 8b,c), indicating that functional similarity of isolates differed from their phylogenetic closeness.

## Conclusion

Taken together, this study represents an important contribution to the literature about microsymbionts nodulating grain legumes in Africa, especially with the evidence provided here on the presence of high genetic diversity of cowpea microsymbionts in Ghanaian and South African soils. Phylogenetic studies of rhizobial isolates from cowpea planted in Ghana and South Africa revealed the presence of potentially novel groups of rhizobia in those environments that are still waiting to be properly identified. The significant correlation between phylogeny and functional genetic distances suggests possible impact of complex host-symbiont dynamics on the stability of the cowpea-*Bradyrhizobium* symbiosis.

## Materials and Methods

### Origin of cowpea root nodules

The nodules used in this study were collected from cowpea plants grown at four locations in the Northern Region (Guinea savanna agroecology) and one location in the Upper East Region (Sudano-sahelian agroecology) of Ghana, and others from two locations, Nelspruit and Klipplaatdrift, respectively, in the lowveld and middleveld areas of the Mpumalanga Province of South Africa (Table 3; Fig. 9). The soil chemical properties as well as geographic coordinates of the sampling sites are shown in Table 3. The nodules used in this study were harvested from either field-grown plants (F) or trapped in the glasshouse (G) using soils collected from the field (Table 3). To trap rhizobia directly in field soils, five cowpea genotypes (Apagbaala, Padi-tuya, Songotra, Omandaw and IT90K-277-2) were planted at Garu, and seven genotypes (Apagbaala, Bawutawuta, Nhyira, Padi-tuya, Songotra, Omandaw and IT90K-277-2) at Kliplaadrift. Rhizobia in soils collected from Nyankpala, Damongo and Nelspruit were trapped in the glasshouse using three cowpea genotypes (Apagbaala, Songotra and IT90K-277-2).

**Table 3:** Origin of cowpea isolates used in this study, and soil chemical properties of the sampling sites.

Country	Region/Province	Location	Latitude	Longitude	pH (H_2_O)	B	C	Ca	Fe	K	Mg	Mn	Na	P	N
					—	(mg.kg^−1^)	(%)	cmol(+).kg^−^	mg.kg^−1^	(mg.kg^−1^)	cmol(+).kg^−1^	(mg.kg^−1^)	(mg.kg^−1^)	(mg.kg^−1^)	(%)
Ghana	Northern	Nyankpala^G^	9.389	−1.006	5.9	0.27	0.40	1.06	59.15	68	0.39	79.22	8	6	0.023
		Damongo^G^	9.043	−1.814	4.3	0.23	0.31	1.13	53.73	90	0.49	54.96	7	10	0.04
	Upper East	Garu^F^	10.929	−0.125	5.3	0.25	0.55	1.74	40.32	38	0.46	39.09	9	5	0.048
South Africa	Mpumalanga	Klipplaatdrift^F^ (Middleveld region)	−25.233	29.033	6.0	0.23	0.30	3.03	36.80	135	1.19	56.14	11	56	0.29
		Nelspruit^G^ (Lowveld region)	−25.474	30.970	4.9	0.09	0.26	0.74	38.16	43	0.38	39.90	12	32	0.029

Note: ^F^Rhizobia trapped by direct planting of Kersting’s bean in the fields, ^G^Rhizobia trapped in the glasshouse from sampled field soils.

### Trapping rhizobia in the field

To trap soil rhizobia under field conditions, cowpea seeds were surface-sterilized in 95% ethanol (3–5 minutes), followed by immersion in NaOCl for 3 minutes, and rinsed 5 times with sterile distilled water. The sterilized seeds of each test cowpea genotype were sown in 3 × 2 m plots with three replications at Garu and Kliplaadrift. After germination, weeds were controlled using hand hoes when necessary. At flowering (at 45 days after planting), 3 plants were dug out per plot and separated into shoots and nodulated roots, placed in pre-labelled brown paper bags and transported to the laboratory, where the roots were gently washed in running tap water to remove soil and adhering debris. Healthy nodules were detached with small root segments and dehydrated on silica gel prior to bacterial isolation.

### Trapping rhizobia in the glasshouse

Where field-planting could not be carried out, soils were sampled from those locations (namely, Nyankpala, Damongo and Nelspruit) for trapping rhizobia under glasshouse conditions using three cowpea genotypes (Apagbaala, Songotra and IT90K-277-2) as host legumes. Two sterilized seeds of each genotype were planted in triplicate pots containing sterile (autoclaved) sand. Soil inocula were prepared by suspending 20 g of each soil sample in 1000 mL of sterile distilled water. Each pot was then inoculated with soil suspension from different locations. The plants were supplied with sterile (autoclaved) N-free nutrient solution^44^, and when necessary, irrigated with sterile distilled water. At flowering, the plants were uprooted and nodules removed.

### Isolation of bacteria from nodules

Bacterial isolation from cowpea root nodules was carried out, as described by Somasegaran and Hoben^45^. Healthy and functional nodules were selected from each source for bacterial isolation. Briefly, the nodules were surface-sterilized by immersion in 75% ethanol for 1 minute, then washed in 3.5% NaOCl for 3 minutes, followed by rinsing five times with sterile distilled water. Each surface-sterilized nodule was then crushed in a loop of sterile distilled water in a sterile petri dish, and the nodule macerate streaked on yeast mannitol agar (YMA) plates, and incubated at 28 °C. Colony appearance was observed from two to twelve days after incubation, and single colonies were selected and streaked onto YMA plates for further characterization.

### Bacterial authentication in the glasshouse

In fulfilment of Koch’s postulates, single-colony cultures were evaluated for their ability to form root nodules on their homologous host^46^. Cowpea genotype Padi-tuya was used to test the nodulation ability of the bacterial isolates. Before planting, seeds were surface-sterilized^46^ and two seeds planted in sterile autoclaved sand contained in sterile pots in a naturally lit glasshouse. The seedlings were thinned to one plant per pot after germination and grown in average temperature of 28 °C in the glasshouse. Four replicate pots were used for each isolate. Six-d-old seedlings were inoculated with 1 mL broth suspension of bacterial culture grown to exponential phase (10^6^–10^7^ cells/ml) using sterile micropipettes. Uninoculated plants and 5 mM NO_3_^−1^-fed plants were included as controls. The inoculated seedlings were fed with sterile N-free nutrient solution^44^ and sterile distilled water in alternation. All the cowpea treated plants were harvested at 50 days after planting (DAP) and assessed for nodulation.

### Gas-exchange studies and symbiotic effectiveness

To assess symbiotic effectiveness of the bacterial isolates, photosynthetic rates (A), stomatal conductance (gs) and leaf transpiration (E) were measured on young and fully expanded trifoliate leaves of each replicate plant at 50 DAP using portable infrared red gas analyzer, version 6.2 (LI 6400XT, Lincoln, Nebraska, USA). The chamber conditions used included photosynthetic flux density of 1000 μmolm^−2^s^−1^, reference CO_2_ concentration of 400 μmolmol^−1^ and flow rate of 500 μmols^−1^. Gas-exchange measurements were carried out between 8:30 am and 12:30 pm. The same leaves used for gas-exchange measurements were plucked for chlorophyll determination. For each leaf sample, chlorophyll was extracted from 6 leaf discs (each with an area of 0.786 cm^2^ and weighing ≈ 19.3 mg) using preheated (65 °C) dimethyl sulfoxide^47^. The absorbance of leaf chlorophyll extracts were measured at 645 nm and 663 nm on a Jenway 7300 spectrophotometer and total chlorophyll calculated using the equations described by Richardson *et al*.^47^. The plants were then uprooted and assessed for nodule number (NN), nodule dry matter (NDM), and shoot dry matter (SDM) after oven-drying at 60 °C for 72 h. The relative effectiveness of isolates (RE) was calculated as the shoot biomass of inoculated plants expressed as a percentage of the shoot biomass of the 5 mM KNO_3_-fed cowpea plants.

### Extraction of bacterial genomic DNA and BOX-PCR fingerprinting

Bacterial genomic DNA was extracted using Sigma’s Bacterial Genomic DNA Kit following the manufacturer’s instructions (GenElute^TM^). DNA integrity was checked on 1% agarose gel stained with ethidium bromide.

The bacterial genomic DNA was subjected to rep-PCR (BOX- PCR) using Box-A1R primer (Table S2). The final volume of the PCR reaction mixture was 25 µL, and contained 1 µL (50–70 ng/µL) genomic DNA, 12.5 µL MyTaq PCR master-mix (2x), 1 µL (10 µM) BOX-A1R primer, and 10.5 mL sterile ultrapure H_2_O. PCR amplification was done in a Thermal cycler (T100 BIORAD, USA) using standard temperature profiles (Table S2). The PCR-amplified products were electrophoresed in 1.2% agarose gel for 6 h, visualized and photographed using gel documentation system (BIO-RAD Gel Doc^TM^ XR+). The sizes of bands were determined using the Image Lab software (Bio-Rad version 4.1). Cluster analysis was carried out with the UPGMA (Unweighted Pair Group Method with Arithmetic mean) algorithm using a trial version of the software Bionumerics 7.6 (with permission obtained from Bionumerics).

### PCR amplification of the 16S rRNA, housekeeping (*atpD, glnII, gyrB, rpoB*) and symbiotic (*nifH* and *nodC*) genes

PCR amplification of 16S rRNA, protein-coding (*atpD, glnII, gyrB* and *rpoB*) and symbiotic (*nifH* and *nodC*) genes were each done in 25 µL reaction volume. The reaction mixture contained 1 µL DNA (50–70 ng/µL), 3 µL of myTaq buffer (5x), 1 µL (10 µM) each of forward and reverse primers of the gene of interest, 0.1 µL Taq polymerase (5U) (Bioline, USA), and 18.9 µL double distilled ultrapure water. The PCR was carried out with Bio-Rad T100 thermal cycler using standard temperature profiles (Table S2). The amplified gene products were confirmed by gel electrophoresis in 1.2% agarose gel stained with ethidium bromide in TAE buffer at 85 V for 1 h.

### Sequencing and phylogenetic analysis

For sequencing, amplified PCR products were purified using PCR Cleanup kit (NEB, USA) and following the manufacturer’s instruction. The purified amplified DNA was sent to Macrogen (Netherlands) for sequencing. Thereafter, the quality of sequences was verified using the software BioEdit 7.0.9.0^48^. The BLASTn program was used to identify closely related species in the NCBI database. Pairwise and multiple sequence alignments were done with CLUSTALW, and phylogenetic trees constructed by means of the maximum likelihood statistical method using MEGA 7 software^49^. The evolutionary history was inferred by using the Maximum Likelihood method based on the Kimura 2-parameter model^50^. The robustness of branching was estimated using 1000 bootstrap replicates^51^.

### Phylogenetic and functional correlation

Quantitative data generated from pair-wise genetic distances obtained from gene phylogeny were compared with parameters of nodule functioning elicited by the cowpea isolates. The pair-wise genetic distances of isolates generated from concatenated genes (*atpD* + *glnII* + *gyrB* + *rpoB*) and *nifH* gene phylogenies were determined using Kimura 2-parameter matrices in MEGA7 software^49^. The genetic distances of the same isolates based on soil chemical properties and symbiotic parameters (namely, nodule number, nodule dry matter, shoot dry matter, photosynthetic rate, leaf transpiration and total chlorophyll) were also determined using NTSYSpc software, version 2.21^52^. The relationship between genetic distances based on gene sequences, soil chemical properties and symbiotic parameters of isolates were explored using correlation and regression analysis by means of the software STATISTICA version 10.0^53^.

### Statistical analysis

The effect of soil factors on the distribution of cowpea-nodulating bradyrhizobia was examined using canonical correspondence analysis (CCA) with vegan (version 2.4–2)^54^ of R software^55^. Here, we determined which soil or environmental factor was frequently related to the distribution of the test cowpea-nodulating bradyrhizobia. The graph analysis was done for only the soil factors that showed significant contribution. The general permutation test was used to assess the statistical significance of the ordination axes.

Quantitative data including nodule number, nodule DM, shoot DM, photosynthetic rate (A), leaf transpiration (E) and total chlorophyll were subjected to normality test by determining the skewness, kurtosis, mean and median values of each dataset (n = 72) using the Data Analysis component in Excel 2016. For each dataset, the mean ≈ median and the skewness and kurtosis values ranged between −1.51 to +0.75 and −0.9 to +0.44, respectively, which are within the range of values (±2) consistent with a normal distribution^56^. The data were then subjected to a 1-way ANOVA using STATISTICA 10.0 program^53^. Where there were significant treatment differences, the Duncan multiple range test was used to separate the means at p ≤ 0.05. Pair-wise genetic distances of concatenated and *nif*H gene sequences, as well as that generated from symbiotic and plant growth data of cowpea isolates were subjected to correlation analysis using STATISTICA 10.0 program.

## Electronic supplementary material

supplementary

## Data Availability

Datasets generated and/or analysed during the current study are available from the corresponding author on reasonable request. Nucleotide sequences have been deposited in NCBI GenBank (accession numbers MH339749 - MH339859).

## References

[1] Foyer CH (2016). Neglecting legumes has compromised human health and sustainable food production. Nat. Plants.

[2] Magrini MB (2016). Why are grain-legumes rarely present in cropping systems despite their environmental and nutritional benefits? Analyzing lock-in in the French agrifood system. Ecol. Econ..

[3] Belane AK, Dakora FD (2012). Elevated concentrations of dietarily-important trace elements and macronutrients in edible leaves and grain of 27 cowpea (*Vigna unguiculata* L. Walp.) genotypes: implications for human nutrition and health. Food Nutr. Sci..

[4] Iqbal A, Khalil IA, Ateeq N, Khan MS (2006). Nutritional quality of important food legumes. Food Chem..

[5] Shevkani K, Kaur A, Kumar S, Singh N (2015). Cowpea protein isolates: Functional properties and application in gluten-free rice muffins. LWT - Food Sci. Technol..

[6] Ba FS, Pasquet RS, Gepts P (2004). Genetic diversity in cowpea [*Vigna unguiculata* (L.) Walp.] as revealed by RAPD markers. Genet. Resour. Crop Evol..

[7] Coulibaly S, Pasquet RS, Papa R, Gepts P (2002). AFLP analysis of the phenetic organization and genetic diversity of *Vigna unguiculata* L. Walp. reveals extensive gene flow between wild and domesticated types. Theor. Appl. Genet..

[8] D’Andrea AC, Kahlheber S, Logan AL, Watson DJ (2007). Early domesticated cowpea (*Vigna unguiculata*) from Central Ghana. Antiquity.

[9] FAO. http://www.fao.org/faostat/en. Accessed on 2018-05-23. (2016).

[10] Wade TK (2014). Eco-geographical diversity of cowpea bradyrhizobia in Senegal is marked by dominance of two genetic types. Syst. Appl. Microbiol..

[11] Singh BB, Ajeigbe HA, Tarawali SA, Fernandez-rivera S, Abubakar M (2003). Improving the production and utilization of cowpea as food and fodder. F. Crop. Res..

[12] Tittonell P, Giller KE (2013). When yield gaps are poverty traps: The paradigm of ecological intensification in African smallholder agriculture. F. Crop. Res..

[13] Chidebe IN, Jaiswal SK, Dakora FD (2018). Distribution and phylogeny of microsymbionts associated with cowpea (*Vigna unguiculata*) nodulation in three agroecological regions of Mozambique. Appl. Environ. Microbiol..

[14] Oldroyd GEDD, Murray JD, Poole PS, Downie JA (2011). The rules of engagement in the legume-rhizobial symbiosis. Annu. Rev. Genet..

[15] Kaschuk G, Kuyper TW, Leffelaar PA, Hungria M, Giller KE (2009). Are the rates of photosynthesis stimulated by the carbon sink strength of rhizobial and arbuscular mycorrhizal symbioses?. Soil Biol. Biochem..

[16] Gyogluu, C., Mohammed, M., Jaiswal, S. K., Kyei-Boahen, S. & Dakora, F. D. Assessing host range, symbiotic effectiveness, and photosynthetic rates induced by native soybean rhizobia isolated from Mozambican and South African soils. *Symbiosis* 1–10, 10.1007/s13199-017-0520-5 (2017). 10.1007/s13199-017-0520-5 PMC6015603 29997418

[17] Peoples MB (2009). The contributions of nitrogen-fixing crop legumes to the productivity of agricultural systems. Symbiosis.

[18] Belane AK, Dakora FD (2010). Symbiotic N_2_ fixation in 30 field-grown cowpea (*Vigna unguiculata* L. Walp.) genotypes in the Upper West Region of Ghana measured using ^15^N natural abundance. Biol. Fertil. Soils.

[19] Jaiswal SK, Beyan SM, Dakora FD (2016). Distribution, diversity and population composition of soybean-nodulating bradyrhizobia from different agro-climatic regions in Ethiopia. Biol. Fertil. Soils.

[20] Grönemeyer JL, Kulkarni A, Berkelmann D, Hurek T, Reinhold-Hurek B (2014). Identification and characterization of rhizobia indigenous to the Okavango region in Sub-Saharan Africa. Appl. Environ. Microbiol..

[21] Pule-Meulenberg, F., Belane, A. K., Krasova-Wade, T. & Dakora, F. D. Symbiotic functioning and bradyrhizobial biodiversity of cowpea (*Vigna unguiculata* L. Walp.) in Africa. *BMC Microbiol*. **10** (2010). 10.1186/1471-2180-10-89 PMC2858033 20331875

[22] Degefu T, Wolde-meskel E, Woliy K, Frostegård Å (2017). Phylogenetically diverse groups of *Bradyrhizobium* isolated from nodules of tree and annual legume species growing in Ethiopia. Syst. Appl. Microbiol..

[23] Tampakaki AP, Fotiadis CT, Ntatsi G, Savvas D (2017). Phylogenetic multilocus sequence analysis of indigenous slow-growing rhizobia nodulating cowpea (*Vigna unguiculata* L.) in Greece. Syst. Appl. Microbiol..

[24] Bejarano A, Ramírez-Bahena MH, Velázquez E, Peix A (2014). *Vigna unguiculata* is nodulated in Spain by endosymbionts of Genisteae legumes and by a new symbiovar (*vignae*) of the genus *Bradyrhizobium*. Syst. Appl. Microbiol..

[25] Marinho R (2017). Symbiotic and agronomic efficiency of new cowpea rhizobia from Brazilian Semi-Arid. Soil Plant Nutr..

[26] De Castro JL (2017). Diversity and efficiency of rhizobia communities from iron mining areas using cowpea as a trap plant. Rev. Bras. Cienc. do Solo.

[27] Zinga MK, Jaiswal SK, Dakora FD (2017). Presence of diverse rhizobial communities responsible for nodulation of common bean (*Phaseolus vulgaris*) in South African and Mozambican soils. FEMS Microbiol. Ecol..

[28] Puozaa DK, Jaiswal SK, Dakora FD (2017). African origin of *Bradyrhizobium* populations nodulating Bambara groundnut (*Vigna subterranea* L. Verdc) in Ghanaian and South African soils. PLoS One.

[29] de Bello F (2017). Decoupling phylogenetic and functional diversity to reveal hidden signals in community assembly. Methods Ecol. Evol..

[30] Cadotte M, Albert CH, Walker SC (2013). The ecology of differences: Assessing community assembly with trait and evolutionary distances. Ecol. Lett..

[31] Shelby N (2016). Plant mutualisms with rhizosphere microbiota in introduced versus native ranges. J. Ecol..

[32] Menna P, Pereira AA, Bangel EV, Hungria M (2009). Rep-PCR of tropical rhizobia for strain fingerprinting, biodiversity appraisal and as a taxonomic and phylogenetic tool. Symbiosis.

[33] Helene LCF, Jakeline RMD, Renan RA, Mariangela H (2017). “*Bradyrhizobium mercantei* sp. nov., a nitrogen-fixing symbiont isolated from nodules of *Deguelia costata* (syn. *Lonchocarpus costatus*). Int. J. Syst. Evol. Microbiol..

[34] Ramírez-Bahena MH (2009). *Bradyrhizobium pachyrhizi* sp. nov. and *Bradyrhizobium jicamae* sp. nov., isolated from effective nodules of *Pachyrhizus erosus*. Int. J. Syst. Evol. Microbiol..

[35] Wang JY (2013). *Bradyrhizobium daqingense* sp. nov., isolated from soybean nodules. Int. J. Syst. Evol. Microbiol..

[36] Guo J (2010). A single origin and moderate bottleneck during domestication of soybean (*Glycine max*): Implications from microsatellites and nucleotide sequences. Ann. Bot..

[37] Grönemeyer JL, Hurek T, Bünger W, Reinhold-Hurek B (2016). *Bradyrhizobium vignae* sp. nov., a nitrogen-fixing symbiont isolated from effective nodules of *Vigna* and *Arachis*. Int. J. Syst. Evol. Microbiol..

[38] Ormeño-Orrillo E (2012). Change in land use alters the diversity and composition of *Bradyrhizobium* communities and led to the introduction of *Rhizobium etli* into the tropical rain forest of Los Tuxtlas (Mexico). Microb. Ecol..

[39] Hungria M, Vargas MATT (2000). Environmental factors affecting N_2_ fixation in grain legumes in the tropics, with an emphasis on Brazil. F. Crop. Res..

[40] Jaiswal SK, Naamala J, Dakora FD (2018). Nature and mechanisms of aluminium toxicity, tolerance and amelioration in symbiotic legumes and rhizobia. Biol. Fertil. Soils.

[41] Chen J (2016). Genetic diversity and distribution of bradyrhizobia nodulating peanut in acid-neutral soils in Guangdong Province. Syst. Appl. Microbiol..

[42] Han LL (2009). Unique community structure and biogeography of soybean rhizobia in the saline-alkaline soils of Xinjiang, China. Plant Soil.

[43] Kaschuk G, Hungria M, Leffelaar PA, Giller KE, Kuyper TW (2010). Differences in photosynthetic behaviour and leaf senescence of soybean *(Glycine ma*x [L.]Merrill) dependent on N2 fixation or nitrate supply. Plant Biol.

[44] Broughton BWJ, Dilworth MJ (1971). Control of leghaemoglobin synthesis in snake beans. Biochem. J..

[45] Somasegaran, P. & Hoben, H. *Handbook for rhizobia: Methods in legume-Rhizobium technology*. (Springer-Verlag, 1994).

[46] Vincent, J. M. A *Manual for the Practical Study of Root-Nodule Bacteria. A Manual for the Practical Study of Root-Nodule Bacteria* **15**, (Blackwell Scientific, 1970).

[47] Richardson AD, Duigan SP, Berlyn GP (2002). An evaluation of noninvasive methods to estimate foliar chlorophyll content. New Phytol..

[48] Hall, T. A. BioEdit: a user-friendly biological sequence alignment editor and analysis program for Windows 95/98/NT. 95–98 (1999).

[49] Kumar S, Stecher G, Tamura K (2016). MEGA7: Molecular Evolutionary Genetics Analysis Version 7. 0 for Bigger Datasets. Mol. Biol. Evol..

[50] Kimura M (1980). A simple method for estimating evolutionary rates of base substitutions through comparative studies of nucleotide sequences. J. Mol. Evol..

[51] Felsenstein J (1985). Confidence limits on phylogenies: an approach using the bootstrap. Evolution (N. Y)..

[52] Rohlf, F. System, NTSYSpc: Numerical taxonomy (2009).

[53] Statsoft Inc. Statistica (data analysis software system). version 10. www.statsoft.com (2011).

[54] Oksanen, J. *et al*. vegan: community ecology package, version 2.4-1, R Foundation for Statistical Computing (2016).

[55] R Core Team. “R: A language and environment for statistical computing, R foundation for Statistical Computing, Vienna, Austria [Internet] (2015).

[56] Debbie, H., Peter, M., Diana, D. & Laurence, T. *Research methods for the Biosciences*. (Oxford University Press, 2016).

