# Supplementary material for: Distribution and correlation between phylogeny and functional traits of cowpea (Vigna unguiculata L. Walp.)-nodulating microsymbionts from Ghana and South Africa
Source: Sci Rep. 2018 Dec 20;8:18006. doi: 10.1038/s41598-018-36324-0 (PMC6302100; doi:10.1038/s41598-018-36324-0)
Supplement: Supplementary file 1 — supplementary [file 41598_2018_36324_MOESM1_ESM.docx]

**Distribution and correlation between phylogeny and functional traits of cowpea (*Vigna unguiculata* L. Walp.)-nodulating microsymbionts from Ghana and South Africa**

Mustapha Mohammed^1^; Sanjay K. Jaiswal^2^; Felix D. Dakora^2^

Table S1 Nucleotide sequence information of cowpea nodulating rhizobia from different locations in Ghana and South Africa

| **Locus** |  | **Nucleotide sequence information** | | | |  |  |
| --- | --- | --- | --- | --- | --- | --- | --- |
|  | **No. of isolates**  **used in analysis** | **Conserved (C)** | **Variables (V)** | **Parsimony-informative (Pi)** | **Singleton (S)** | **Total** | **Frequency T/C/A/G (%)** |
| 16S rRNA | 67 | 427 (76.1) | 121 (21.6) | 21 (3.7) | 100 (17.8) | 561 | 20.4/21.9/25.5/32.1 |
| *atp*D | 59 | 91 (37.0) | 153 (32.2) | 57 (23.2) | 96 (39.0) | 246 | 19.9/31.5/15.3/33.3 |
| *gln*II | 67 | 289 (60.5) | 189 (39.5) | 151 (31.6) | 38 (7.9) | 478 | 17.2/33.2/19.4/30.3 |
| *gyr*B | 57 | 174 (32.6) | 350 (65.7) | 173 (32.5) | 177 (33.2) | 533 | 20.1/31.9/16.4/32.6 |
| *rpo*B | 58 | 166 (52.9) | 148 (47.1) | 91 (29.0) | 57 (18.2) | 314 | 16.3/32.2/20.5/31.0 |
| *nif*H | 64 | 110 (54.7) | 91 (45.3) | 77 (38.3) | 14 (7.0) | 201 | 18.9/27.3/19.4/34.4 |
| *nod*C | 51 | 15 (7.8) | 177 (92.2) | 167 (87.0) | 10 (5.2) | 192 | 17.1/27.3/23.6/32.0 |
| *atp*D-*gln*II-*gyr*B-*rpo*B | 40 | 761 (48.4) | 799 (50.9) | 408 (26.0) | 319 (20.3) | 1571 | 18.4/31.9/18.0/31.7 |
| *atp*D-*gln*II-*rpo*B | 46 | 567 (54.6) | 469 (45.2) | 278 (26.8) | 191 (18.4) | 1038 | 17.6/32.5/18.7/31.2 |

Table S2 Primers and temperature profiles used in PCR amplification of genes in this study

| **Primers** | **Sequences 5’ – 3’** | **Temperature profiles** | | **References** |
| --- | --- | --- | --- | --- |
| BOXA1R | CTACGGCAAGGCGACGCTGACG | 7 min at 95°C; 34 x 1 min at 94°C, 1 min at 52.8°C, and 8 min at 65^o^C; 16 min at 65^o^C | | ^1^ |
|  |  |  | |  |
| 16S rRNA F  16S rRNA R | 27-5’AGAGTTTGATCCTGGCTCAG3’-46  1492-5’TACGGTTACCTTGTTACGACTT3’-1471 | 5 min at 95°C, 35 X (1 min at 95°C, 1 min at 55°C, 1 min at 72°C), 10 min at 72°C | | ^2^ |
|  |  | | | |
| *nif*H F  *nif*H R | 28-3’TACGGNAARGGSGGNATCGGCAA3’-50  808-5’AGCATGTCYTCSAGYTCNTCCA3’-787 | 5 min at 94°C, 20 X [30s at 94°C, 30s at 65°C (-0.5°C\cycle), 90s at 72°C], 25 x (30s at 94°C, 30s at 55°C, 90s at 72°C), 10 min at 72°C | | ^3^ |
|  |  | | | |
| *gln*ll F  *gln*II R | 13-5’AAGCTCGAGTACATCTGGCTCGACGG3’-38  681-5’SGAGCCGTTCCAGTCGGTGGTGTCG3’-660 | | 2 min at 95°C, 35 X (45s at 95°C, 30s at 65°C, 90s at 72°C), 10 min at 72°C | ^4^ |
|  |  | | | |
| *gyr*B F  *gyr*B R | 343-5’TTCGACCAGAAYTCCTAYAAGG3’-364  1043-5’AGCTTGTCCTTSGTCTGCG3’-1025 | 10 min at 95°C, 35 X (30s at 94°C, 30s at 58°C, 1 min at 72°C), 10 min at 72°C | | ^5^ |
|  |  | | | |
| *atp*D F  *atp*D R | 189-5’TCTGGTCCGYGGCCAGGAAG3’-208  804-5’CGACACTTCCGARCCSGCCTG3’-784 | 2 min at 95^o^C, 35 x (45s at 95^o^C, 30s at 65^o^C, 1.5min at 72^o^C), 10 mins at 72^o^C | | ^4^ |
|  |  | | | |
| *nod*C F  *nod*C R | 5’GTC GAT TGC MRG TCA AGA CTA CG3’  5’GCC AGG TCT IGT TGC GAT TGC TC3’ | 30s at 94°C, 40× [30s at 94°C, 1 min at 55.4°C, 30s at 72°C], 5 min 72°C | | ^6^ |
|  |  | | | |
| *rpo*B F  *rpo*B R | 575-5’ACATCGAGTTCGACGCCAAGG3’-595  1054-5’CATTGACGTGGTCGATGTCG3’-1035 | 5 min at 95°C; 20 x 45s at 95°C, 30s at 60°C (-0.5°C per cycle) and 1 min 30s at 72°C; 25 x 30s at 94°C, 30s at 55°C, 1 min 30s at 72°C; 10 min at 72°C | | ^3^ |

References

1. Versalovic, J., Schneider, M., De Bruihn, F. J. & Lupski, J. R. Genomic fingerprinting of bacteria using Repetitive Sequence-Based ploymerase chain reaction. *J. Clin. Microbiol.* **5,** 25–40 (1994).

2. Willems, A. et al. DNA – DNA hybridization study of *Bradyrhizobium* strains. *Int. J. Syst. Evol. Microbiol.* **51,** 1315–1322 (2001).

3. Nzoué, A. et al. Multilocus sequence analysis of bradyrhizobia isolated from *Aeschynomene* species in Senegal. Syst. Appl. Microbiol. 32, 400–412 (2009).

4. Stepkowski, T. et al. *Bradyrhizobium canariense* and *Bradyrhizobium japonicum* are the two dominant rhizobium species in root nodules of lupin and serradella plants growing in Europe. *Syst. Appl. Microbiol.* **34,** 368–375 (2011).

5. Marek-Kozaczuk, M., Leszcz, A., Wielbo, J., Wdowiak-Wróbel, S. & Skorupska, A. *Rhizobium pisi* sv. trifolii K3.22 harboring nod genes of the *Rhizobium leguminosarum* sv. trifolii cluster. *Syst. Appl. Microbiol.* **36,** 252–258 (2013).

6. Laguerre, G. et al. Classification of rhizobia based on nodC and nifH gene analysis reveals a close phylogenetic relationship among *Phaseolus vulgaris* symbionts. *Microbiology* **147,** 981–993 (2001).

**TUTVUSA31**

*Bradyrhizobium brasilense* UFLA03-321^T^ (KF452730.1)

**TUTVUSA44**

**TUTVUSA33**

**TUTVUSA36**

*Bradyrhizobium elkanii* USDA 76^T^ (AY386758)

*Bradyrhizobium pachyrhizi* PAC48^T^ (FJ428208)

*Bradyrhizobium tropiciagri* SEMIA 6148^T^ (FJ390968)

*Bradyrhizobium embrapense* SEMIA 6208^T^ (HQ634875)

*Bradyrhizobium macuxiense* BR 10303^T^ (LNCU01000024.1)

**TUTVUGH6**

**TUTVUGH17**

**TUTVUGH18**

**TUTVUGH22**

**TUTVUGH24**

*Bradyrhizobium ingae* BR 10250^T^ (KY753593.1)

*Bradyrhizobium denitrificans* LMG 8443^T^ (FM253153.1)

*Bradyrhizobium iriomotense*^T^ (AB300994.1)

*Bradyrhizobium yuanmingense* CCBAU 10071^T^ (AY386760)

*Bradyrhizobium forestalis* INPA54B^T^ (KF452722.1)

*Bradyrhizobium subterraneum* 58 2-1^T^ (KX661391.1)

*Bradyrhizobium betae* LMG 21987^T^ (FM253129.1)

*Bradyrhizobium shewense* ERR11^T^ (NZ FMAI01000019.1)

*Bradyrhizobium ottawaense* OO99^T^ (HQ455212)

*Bradyrhizobium japonicum* E109^T^ (CP010313.1)

*Bradyrhizobium diazoefficiens* USDA 110^T^ (CP011360.1)

*Bradyrhizobium guangdongense* CCBAU 51649^T^ (KC508916)

*Bradyrhizobium ganzhouense* RITF807^T^ (JX277183)

*Bradyrhizobium americanum* CMVU44^T^ (KC247125.1)

**TUTVUSA28**

*Bradyrhizobium daqingense* CCBAU 15774^T^ (HQ231289)

*Bradyrhizobium liaoningense* bv. glycinearum LMG 18230 (AY386752)

*Bradyrhizobium mercantei* SEMIA 6399^T^ (NZ MKFI01000006.1)

*Bradyrhizobium sacchari* BR10280^T^ (KX065107.1)

*Bradyrhizobium arachidis* CCBAU 45332^T^ (JQ011347.1)

**TUTVUGH25**

*Bradyrhizobium huanghuaihaiense* CCBAU 23303^T^ (HQ231682)

*Bradyrhizobium guangxiense* CCBAU 53363^T^ (KC508926)

*Bradyrhizobium rifense* CTAW71^T^ (GU001617)

*Bradyrhizobium canariense* LMG 22265^T^ (FM253135)

*Bradyrhizobium lupini* USDA 3051^T^ (KU738808.1)

*Bradyrhizobium cytisi* CTAW11^T^ (GU001613)

*Bradyrhizobium centrosematis* A9^T^ (KC247129.1)

**TUTVUSA50**

**TUTVUSA48**

*Bradyrhizobium jicamae* PAC68^T^ (FJ428211)

**TUTVUSA43**

**TUTVUSA41**

**TUTVUSA45**

*Bradyrhizobium namibiense* 5-10^T^ (KX661387.1)

*Bradyrhizobium paxllaeri* LMTR 21^T^ (KF896186)

*Bradyrhizobium kavangense* 14-3^T^ (KY753592.1)

*Bradyrhizobium oligotrophica* LMG 10732^T^ (JQ619232)

*Bradyrhizobium icense* LMTR 13^T^ (KF896192)

*Bradyrhizobium lablabi* CCBAU 23086^T^ (GU433473)

*Bradyrhizobium retamae* Ro19^T^ (KC247101)

*Bradyrhizobium algeriense* RST89^T^ (KF956544.1)

*Bradyrhizobium valentinum* LmjM3^T^ (JX518561)

*Rhizobium lusitanum* p1-7^T^ (DQ431671)

94

99

98

81

65

87

55

58

51

52

0.1

**I**

**II**

**III**

**IV**

**V**

Figure S1. Maximum likelihood molecular phylogenetic analysis of cowpea nodulating rhizobia from Ghana and South Africa based on *atp*D gene sequences. The evolutionary history was inferred by using the maximum likelihood method based on the Kimura 2-parameter model^50^. The tree is drawn to scale, with branch lengths measured in the number of substitutions per site. The analysis involved 59 nucleotide sequences. Codon positions included were 1st+2nd+3rd+Noncoding. All positions containing gaps and missing data were eliminated. Evolutionary analyses were conducted in MEGA7^49^.

*Bradyrhizobium canariense* bv. genistearum BTA-1^T^ (AY386765.1)

*Bradyrhizobium lupini* USDA 3051 (KM114862)

*Bradyrhizobium iriomotense*^T^ (AB300995.1)

*Bradyrhizobium ingae* BR 10250 (KF927067)

*Bradyrhizobium ganzhouense* RITF807^T^ (JX277111)

*Bradyrhizobium cytisi* CTAW11^T^ (GU001594)

*Bradyrhizobium rifense* CTAW71^T^ (GU001604)

*Bradyrhizobium stylosanthis* BR 446^T^ (KU724148.1)

*Bradyrhizobium huanghuaihaiense* CCBAU 23303^T^ (HQ231639)

*Bradyrhizobium japonicum*^T^ (AF169582)

*Bradyrhizobium diazoefficiens* USDA 110^T^ (CP011360.1)

*Bradyrhizobium betae* LMG 21987^T^ (AB353733.1)

*Bradyrhizobium liaoningense* bv. glycinearum LMG 18230^T^ (AY386775)

*Bradyrhizobium shewense* ERR11^T^ (JQ809893.1)

*Bradyrhizobium ottawaense* OO99^T^ (HQ587750)

*Bradyrhizobium cajani* AMBPC1010^T^ (KY349442.1)

**TUTVUSA28**

*Bradyrhizobium daqingense* CCBAU 15774^T^ (HQ231301)

*Bradyrhizobium americanum* CMVU44^T^ (KX012942)

**TUTVUSA43**

**TUTVUSA48**

**TUTVUSA50**

**TUTVUSA41**

**TUTVUSA45**

*Bradyrhizobium arachidis* CCBAU 051107^T^ (HM107251)

*Bradyrhizobium centrolobii* BR 10245^T^ (KX527991.1)

*Bradyrhizobium neotropicale* BR 10247^T^ (KJ661700.1)

*Bradyrhizobium yuanmingense* CCBAU 10071^T^ (AY386780)

*Bradyrhizobium subterraneum* 60 2-1^T^ (KM378485.1)

**TUTVUGH25**

*Bradyrhizobium vignae* 7-2^T^ (KM378443)

*Bradyrhizobium kavangense* 14-3^T^ (KM378446)

*Bradyrhizobium guangdongense* CCBAU 51649^T^ (KC509023)

*Bradyrhizobium centrosematis* A9^T^ (KX012940.1)

*Bradyrhizobium guangxiense* CCBAU 53363^T^ (KC509033)

*Bradyrhizobium manausense* BR 3351^T^ (KF785986)

*Bradyrhizobium macuxiense* BR 10303^T^ (KX527995.1)

*Bradyrhizobium sacchari* BR 10303^T^ (KX527995.1)

*Bradyrhizobium erythrophlei* CCBAU 53325^T^ (KF114693)

*Bradyrhizobium mercantei* 6399^T^ (KX690621.1)

**TUTVUGH2**

*Bradyrhizobium elkanii* USDA 76^T^ (AY599117)

*Bradyrhizobium tropiciagri* SEMIA 6148^T^ (FJ391048)

*Bradyrhizobium embrapense* SEMIA 6208^T^ (GQ160500)

**TUTVUGH18**

**TUTVUGH24**

**TUTVUGH22**

**TUTVUGH6**

**TUTVUGH17**

*Bradyrhizobium viridifuturi* SEMIA 690^T^ (KR149131)

*Bradyrhizobium ferriligni* CCBAU 51502^T^ (KJ818099)

**TUTVUSA31**

**TUTVUSA36**

**TUTVUSA33**

**TUTVUSA44**

*Bradyrhizobium pachyrhizi* PAC48 (FJ428201)

*Bradyrhizobium valentinum* LmjM3^T^ (JX518575)

*Bradyrhizobium algeriense* RST89^T^ (FJ264924.1)

*Bradyrhizobium lablabi* CCBAU 23086^T^ (GU433498)

*Bradyrhizobium paxllaeri* LMTR 21^T^ (KF896169)

*Bradyrhizobium jicamae* PAC68^T^ (FJ428204)

*Bradyrhizobium icense* LMTR 13^T^ (KF896175)

*Bradyrhizobium retamae* Ro19^T^ (KC247108)

*Bradyrhizobium namibiense* 5-10^T^ (KM378440.1)

*Bradyrhizobium oligotrophica* LMG 10732^T^ (JQ619233)

*Bradyrhizobium denitrificans* LMG 8443^T^ (HM047121)

*Rhizobium lusitanum* P1-7^T^ (EF639841.1)

73

98

97

98

96

66

93

99

89

52

54

88

80

67

98

75

83

97

50

99

98

92

99

90

91

97

63

86

85

78

50

57

97

0,02

**I**

**II**

**III**

**IV**

**V**

**VI**

Figure S2. Maximum likelihood molecular phylogenetic analysis of cowpea nodulating rhizobia from Ghana and South Africa based on *gln*II gene sequences. The evolutionary history was inferred by using the maximum likelihood method based on the Kimura 2-parameter model^50^. The tree is drawn to scale, with branch lengths measured in the number of substitutions per site. The analysis involved 67 nucleotide sequences. Codon positions included were 1st+2nd+3rd+Noncoding. All positions containing gaps and missing data were eliminated. Evolutionary analyses were conducted in MEGA7^49^.

*Bradyrhizobium paxllaeri* LMTR 21^T^ (KF896195)

*Bradyrhizobium lablabi* CCBAU 23086^T^ (KF962696)

*Bradyrhizobium jicamae* PAC 68^T^ (HQ873309)

*Bradyrhizobium retamae* Ro19^T^ (KF962698)

*Bradyrhizobium icense* LMTR 13^T^ (KF896201)

*Bradyrhizobium namibiense* 5-10^T^ (KX661393.1)

*Bradyrhizobium erythrophlei* CCBAU 53325^T^ (KF114717)

*Bradyrhizobium algeriense* RST91^T^ (NZ PYCN01000001.1)

*Bradyrhizobium valentinum* LmjM3^T^ (LLXX01000044.1)

**TUTVUGH2**

**TUTVUSA44**

*Bradyrhizobium elkanii* LMG 6134^T^ (AM418800.1)

*Bradyrhizobium pachyrhizi* PAC 48^T^ (HQ873310)

**TUTVUSA31**

*Bradyrhizobium brasilense* UFLA03-321^T^ (KF452827.1)

**TUTVUSA33**

**TUTVUSA36**

*Bradyrhizobium embrapense* SEMIA 6208^T^ (HQ634891)

**TUTVUGH18**

**TUTVUGH24**

**TUTVUGH17**

*Bradyrhizobium tropiciagri* SEMIA 6148^T^ (HQ634890)

*Bradyrhizobium viridifuturi* SEMIA 690^T^ (KR149134)

*Bradyrhizobium ferriligni* CCBAU 51502^T^ (KJ818102)

*Bradyrhizobium mercantei* SEMIA 6399^T^ (KX690623.1)

*Bradyrhizobium sacchari* BR 10303^T^ (KX528008.1)

*Bradyrhizobium liaoningense* LMG 18230^T^ (FM253223.1)

*Bradyrhizobium shewense* ERR11^T^ (NZ FMAI01000013.1)

*Bradyrhizobium ottawaense* OO99^T^ (HQ873179)

Bradyrhizobium diazoefficiens USDA 110^T^ (CP011360.1)

*Bradyrhizobium betae* LMG 21987^T^ (FM253217.1)

*Bradyrhizobium canariense* LMG 22265^T^ (FM253220.1)

*Bradyrhizobium rifense* CTAW71^T^ (KC569466)

*Bradyrhizobium ganzhouense*^T^ (KP420022)

*Bradyrhizobium cytisi* CTAW11^T^ (KF532653)

*Bradyrhizobium denitrificans* LMG 8443^T^ (FM253239.1)

*Bradyrhizobium subterraneum* 58 2-1^T^ (KX661396.1)

*Bradyrhizobium forestalis* INPA54B^T^ (KF452831.1)

**TUTVUGH25**

*Bradyrhizobium iriomotense*^T^ (AB300997.1)

*Bradyrhizobium centrolobii* BR 10245^T^ (KX528004.1)

*Bradyrhizobium ingae* BR 10250^T^ (KF927079)

*Bradyrhizobium kavangense* 14-3^T^ (KX661397.1)

*Bradyrhizobium huanghuaihaiense* CCBAU 23303^T^ (KF962695)

*Bradyrhizobium manausense* BR 3351^T^ (KF786000)

**TUTVUSA28**

*Bradyrhizobium daqingense* CCBAU 15774^T^ (KF962694)

*Bradyrhizobium yuanmingense* LMG 21827^T^ (FM253226.1)

*Bradyrhizobium guangxiense* CCBAU 53363^T^ (KC509082)

**TUTVUSA41**

**TUTVUSA45**

**TUTVUSA43**

**TUTVUSA48**

*Bradyrhizobium guangdongense* CCBAU 51649^T^ (KC509072)

*Bradyrhizobium stylosanthis* BR 446^T^ (KU724151.1)

*Bradyrhizobium japonicum* LMG 6138^T^ (AM418801.1)

*Rhizobium lusitanum* strain P1-7^T^ (KC293525.1)

97

99

93

93

91

100

98

98

99

56

92

91

59

93

89

89

73

92

66

63

61

0.1

**I**

**II**

**III**

**IV**

**V**

Figure S3. Maximum likelihood molecular phylogenetic analysis of cowpea nodulating rhizobia from Ghana and South Africa based on *gyr*B gene sequences. The evolutionary history was inferred by using the maximum likelihood method based on the Kimura 2-parameter model^50^. The percentage of trees in which the associated taxa clustered together is shown next to the branches. The tree is drawn to scale, with branch lengths measured in the number of substitutions per site. The analysis involved 57 nucleotide sequences. Codon positions included were 1st+2nd+3rd+Noncoding. All positions containing gaps and missing data were eliminated. Evolutionary analyses were conducted in MEGA7^49^.

**TUTVUSA45**

**TUTVUSA50**

**TUTVUSA41**

**TUTVUSA43**

**TUTVUSA48**

**TUTVUSA28**

*Bradyrhizobium daqingense* CCBAU 15774^T^ (JX437676.1)

*Bradyrhizobium shewense* ERR11^T^ (FMAI01000007.1)

*Bradyrhizobium arachidis* CCBAU 051107^T^ (JX437682.1)

*Bradyrhizobium guangxiense* CCBAU 53363^T^ (KC509328.1)

**TUTVUGH25**

*Bradyrhizobium vignae* 7-2^T^ (KM378308.1)

*Bradyrhizobium liaoningense* LMG 18230^T^ (EF190181.1)

*Bradyrhizobium forestalis* INPA54B^T^ (PGVG01000026.1)

*Bradyrhizobium yuanmingense* LMG 21827^T^ (FM253269.1)

*Bradyrhizobium subterraneum* 54 1-1^T^ (KM378344.1)

*Bradyrhizobium ottawaense* OO99^T^ (HQ587518.1)

*Bradyrhizobium centrolobii* BR 10245^T^ (KF983827.3)

*Bradyrhizobium neotropicale* BR 10247^T^ (KF983829.2)

*Bradyrhizobium iriomotense* LMG 24129^T^ (HQ587646.1)

*Bradyrhizobium ingae* BR 10250^T^ (KF927073.1)

*Bradyrhizobium guangdongense* CCBAU 51649^T^ (KC509318.1)

*Bradyrhizobium stylosanthis* BR 446^T^ (KU724166.1)

*Bradyrhizobium huanghuaihaiense* CCBAU 23303^T^ (JX437679.1)

*Bradyrhizobium manausense* BR 3351^T^ (KF785998.1)

*Bradyrhizobium diazoefficiens* SEMIA 6059^T^ (JX867244.1)

*Bradyrhizobium cytisi* LMG 25866^T^ (JN186288.1)

*Bradyrhizobium rifense* CTAW71^T^ (KF962715.1)

*Bradyrhizobium canariense* LMG 22265^T^ (FM253263.1)

*Bradyrhizobium betae* LMG 21987^T^ (FM253260.1)

*Bradyrhizobium japonicum* USDA 6 (LC167354.1)

*Bradyrhizobium kavangense* 14-3^T^ (KM378311.1)

*Bradyrhizobium namibiense* 5-10^T^ (KM378306.1)

*Bradyrhizobium lablabi* CCBAU 23086^T^ (JX437677.1)

*Bradyrhizobium paxllaeri* LMTR 21^T^ (KP308154.1)

*Bradyrhizobium jicamae* LMG 24556^T^ (HQ587647.1)

*Bradyrhizobium icense* LMTR 13^T^ (CP016428.1)

*Bradyrhizobium retamae* Ro19^T^ (KF962714.1)

*Bradyrhizobium algeriense* RST91^T^ (NZ PYCN01000011.1)

*Bradyrhizobium erythrophlei* CCBAU53325^T^ (MG811654.1)

*Bradyrhizobium macuxiense* BR 10303^T^ (LNCU01000011.1)

**TUTVUGH18**

**TUTVUGH22**

**TUTVUGH24**

**TUTVUGH6**

**TUTVUGH17**

*Bradyrhizobium embrapense* SEMIA 6208^T^ (HQ634910.1)

**TUTVUGH2**

*Bradyrhizobium viridifuturi* SEMIA 690^T^ (KU724169.1)

*Bradyrhizobium elkanii* USDA 76^T^ (EF190188.1)

**TUTVUSA36**

**TUTVUSA44**

*Bradyrhizobium brasilense* UFLA03-321^T^ (KF452879.1)

*Bradyrhizobium ferriligni* CCBAU51502^T^ (MG811655.1)

*Bradyrhizobium pachyrhizi* PAC48^T^ (LM994172.1)

*Bradyrhizobium oligotrophicum* S58 LMG 10732^T^ (KF962713.1)

*Bradyrhizobium denitrificans* LMG 8443^T^ (FM253282.1)

*Rhizobium lusitanum* P1-7 (FJ816277.1)

55

86

57

86

72

50

74

63

69

95

62

91

97

73

62

67

90

83

91

99

79

72

85

83

70

77

0,05

**I**

**II**

**III**

**IV**

Figure S4. Maximum likelihood molecular phylogenetic analysis of cowpea nodulating rhizobia from Ghana and South Africa based on *rpo*B gene sequences. The evolutionary history was inferred by using the maximum likelihood method based on the Kimura 2-parameter model^50^. The percentage of trees in which the associated taxa clustered together is shown next to the branches. The tree is drawn to scale, with branch lengths measured in the number of substitutions per site. The analysis involved 58 nucleotide sequences. Codon positions included were 1st+2nd+3rd+Noncoding. All positions containing gaps and missing data were eliminated. Evolutionary analyses were conducted in MEGA7^49^.


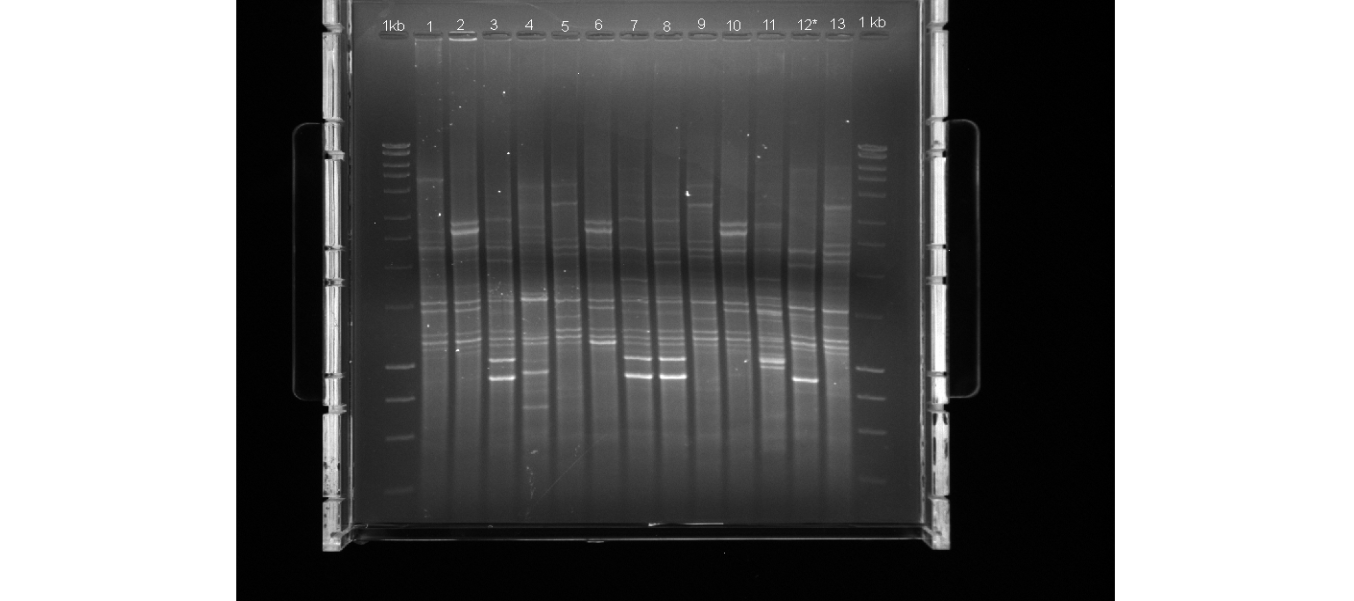

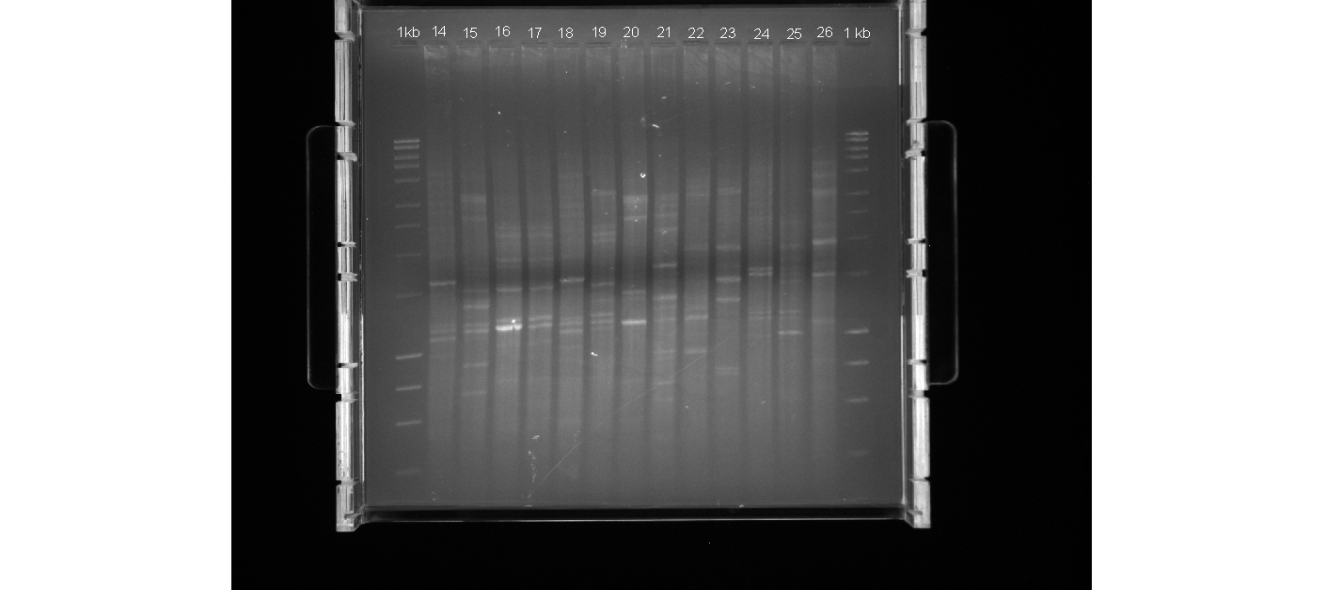

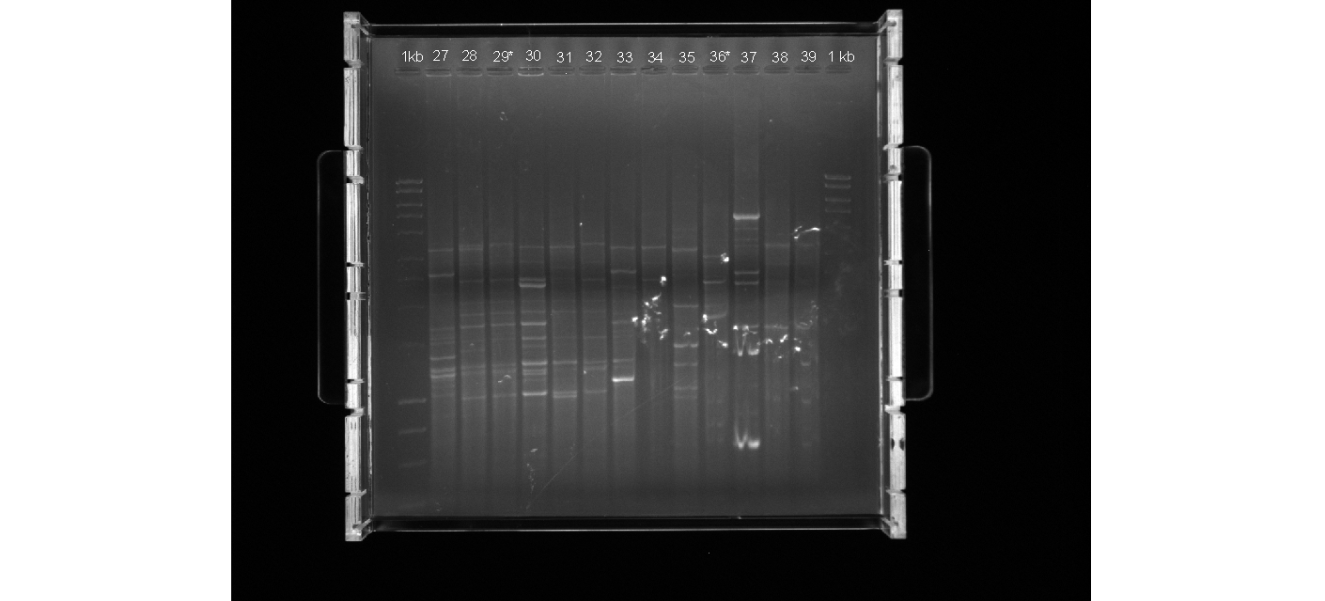

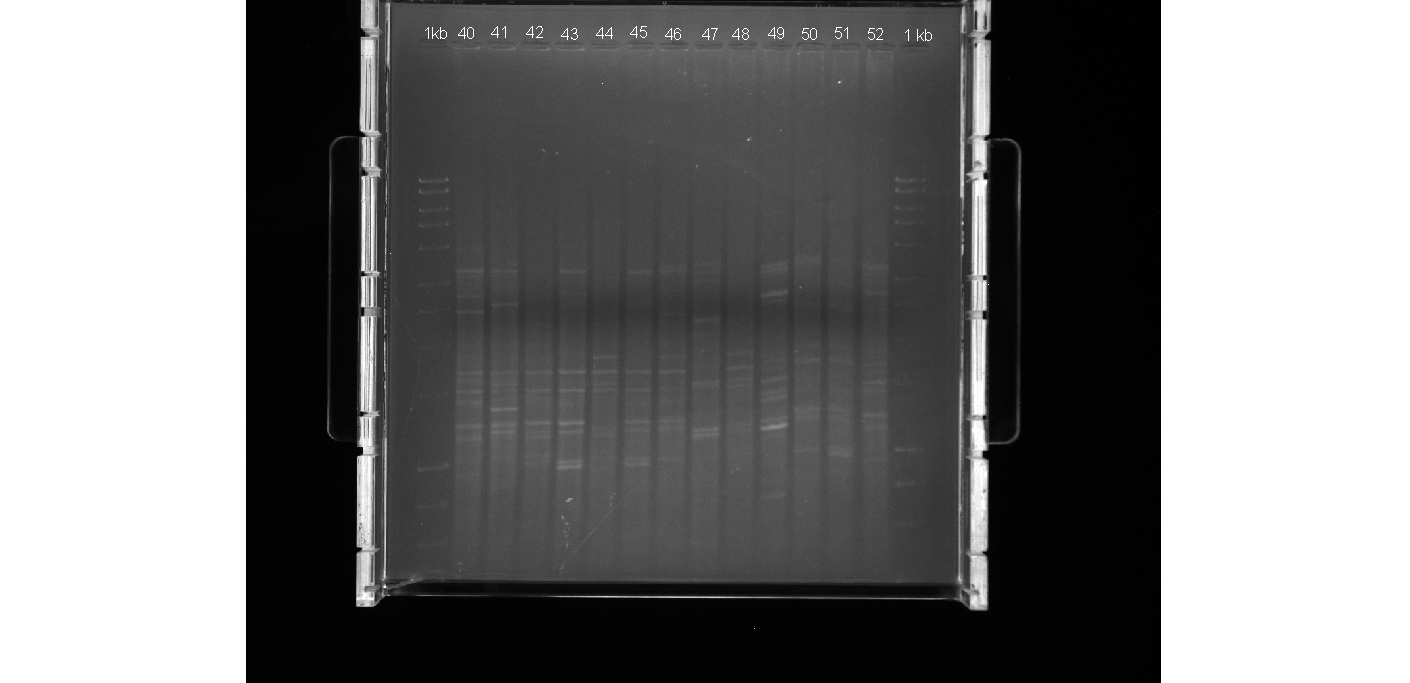

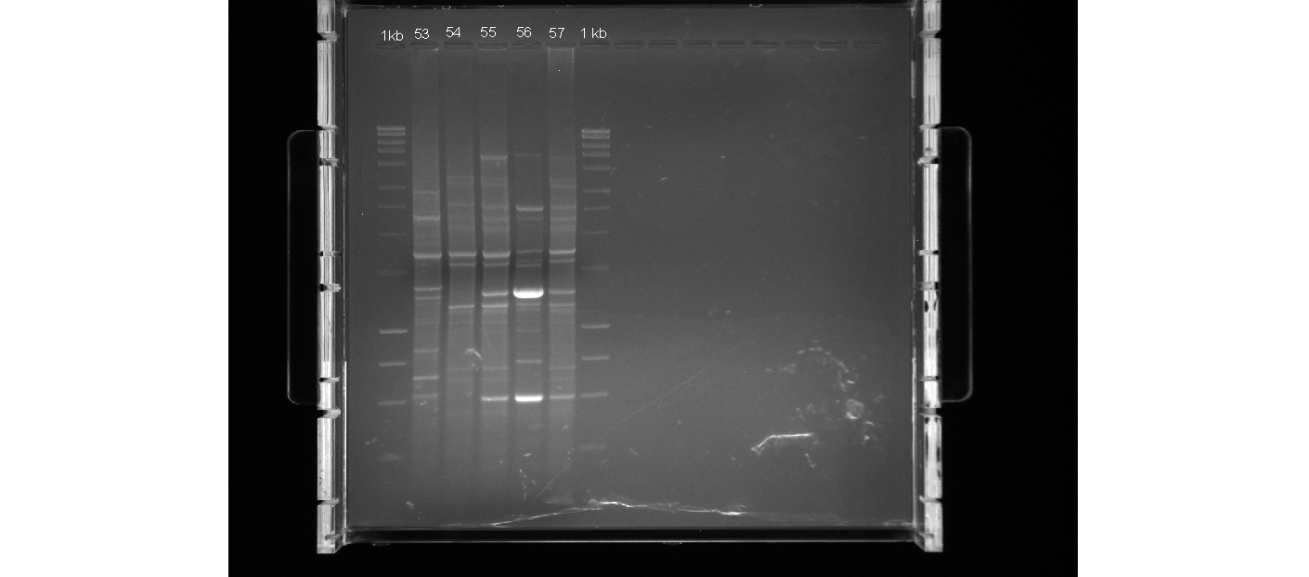


Figure S5. Gel images showing the Box-PCR profiles of 57 cowpea nodulating rhizobia from different locations in Ghana and South Africa. The PCR-amplified products were electrophoresed in 1.2% agarose gel (20 × 15 cm gel size) for 6 h at 85 v per gel. In the gel picture, the isolates are serially numbered as: 1-TUTVUGH6, 2- TUTVUGH7, 3-TUTVUGH8, 4-TUTVUGH9, 5-TUTVUGH10, 6-TUTVUGH11, 7-TUTVUGH12, 8-TUTVUGH13, 9-TUTVUGH14, 10-TUTVUGH15, 11-TUTVUGH16, 12*-TUTVUGH20, 13-TUTVUGH18, 14- TUTVUGH19, 15-TUTVUGH20, 16-TUTVUGH21, 17-TUTVUGH22, 18-TUTVUGH23, 19-TUTVUGH24, 20-TUTVUGH25, 21-TUTVUGH26, 22-TUTVUGH27, 23-TUTVUGH1, 24-TUTVUGH2, 25-TUTVUGH3, 26-TUTVUGH, 27-TUTVUSA50, 28-TUTVUSA51, 29*-TUTVUSA52, 30-TUTVUSA53, 31-TUTVUSA54, 32-TUTVUSA55, 33-TUTVUSA56, 34-TUTVUSA57, 35-TUTVUSA28, 36*-TUTVUSA29, 37-TUTVUSA30, 38-TUTVUSA31, 39-TUTVUSA32, 40-TUTVUSA37, TUTVUSA38-41, 42-TUTVUSA39, 43-TUTVUSA40, 44-TUTVUSA41, 45-TUTVUSA42, 46-TUTVUSA43, 47-TUTVUSA44, 48-TUTVUSA45, 49-TUTVUSA46, 50-TUTVUSA47, 51-TUTVUSA48, 52-TUTVUSA49, 53-TUTVUGH5, 54-TUTVUSA33, 55-TUTVUSA34, 56-TUTVUSA35, 57-TUTVUSA36 and 1kb-ladder. NB: *isolates were not included in the Box-PCR cluster analysis.
